# Rapid and catalyst free synthesis of new bis(benzo[*g*]chromene) and bis(pyrano[3,2-*c*]chromene) derivatives and optimization of reaction conditions using response surface methodology†

**DOI:** 10.1039/c9ra07809f

**Published:** 2019-12-02

**Authors:** Fahimeh Sadat Hosseini, Mohammad Bayat, Milad Afsharnezhad

**Affiliations:** Department of Chemistry, Faculty of Science, Imam Khomeini International University Qazvin Iran bayat_mo@yahoo.com m.bayat@sci.ikiu.ac.ir +98 28 33780040

## Abstract

4,4′-(1,4-phenylene)bis(2-(alkylamino)-3-nitro-4*H*-benzo[*g*]chromene-5,10-dione) and 4,4′-(1,4-phenylene)bis(2-(alkylamino)-3-nitropyrano[3,2-*c*]chromen-5(4*H*)-one) derivatives are synthesized by a one-pot, multi-component reaction of *N*-alkyl-1-(methylthio)-2-nitroethenamine (derived from the reaction of various amines and 1,1-bis(methylthio)-2-nitroethene) with terephthalaldehyde or isophthalaldehyde, and 2-hydroxy-1,4-naphthoquinone or 4-hydroxycoumarin in EtOH/H_2_O (85 : 15) as the solvent at 89 °C. Response surface methodology (RSM) is used to investigate the effect of reaction temperature and water content of aqueous ethanol on the product yields and reaction time. The notable features of this work are the optimization of reaction conditions with minimal experiments, absence of catalyst, good yields, simple work-up and the non-chromatographic purification of products.

## Abstract 2

One-pot synthesis of C_2_-symmetric chromene derivatives, which serve as building blocks to prepare liquid crystals with potential electrical or optical properties.

## Graphical abstract

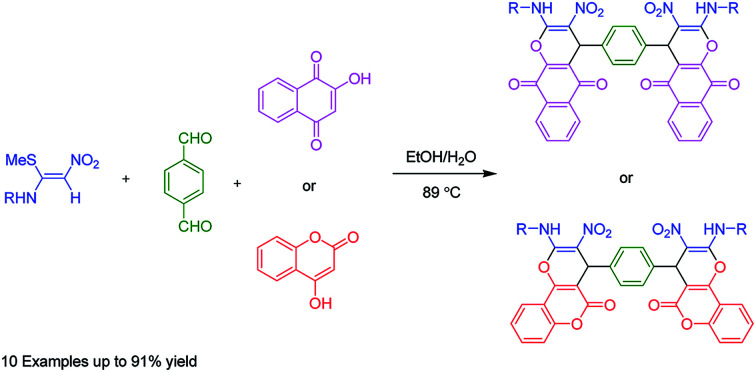

## Introduction

Response surface methodology (RSM), is one of the most commonly used experimental designs for optimizing process conditions.^1–7^ Traditionally, optimization has been carried out by monitoring the influence of one factor at a time on an experimental response.^8^ RSM is a more economical method because a small number of experiments are carried out for monitoring the interaction of the independent variables in the response. In conventional optimization, the increase in the number of experiments necessary to perform the research, leads to an increase in time, expense, and the consumption of reagents and materials for experiments.^8,9^ Hence, response surface methodology (RSM) may be considered as an efficient way to deal with the limitations of the conventional method.^10^ In the recent years, several studies used successfully RSM for optimizing reaction conditions such as optimization of reaction parameters for the synthesis of chromium methionine complex (Xiao *et al.*, 2013),^5^ optimization of the synthesis of highly functionalized pyrimido[1,2-*b*]indazoles *via* 6-*endo-dig* cyclization (Roopan *et al.*, 2016),^11^ and optimization of the reaction conditions for the synthesis of 1-(2-aminoethyl)-2-imidazolidone (Cai *et al.*, 2017).^12^

Multi-component reactions (MCRs) represent a powerful and efficient method for construction of complex molecules with biological potential.^13^ They offer remarkable advantages like convergence, operational simplicity, facile automation, reduction in the number of workups, extraction and purification processes.^14^ Therefore, the design of novel MCRs has attracted great attention from synthetic organic chemists.^15–20^

Chromenes and their fused analogues are a group of biologically active molecules occurring extensively in nature with a wide range of molecular modifications.^21^ Among them benzochromenes are one of the privileged scaffolds with a medicinal pharmacophore such as antibacterial,^22^ antimicrobial,^23^ antitumor,^24^ and anti-inflammatory.^25–28^ Also pyranochromenes are an important and essential category of chromenes and have extensive bioactivities, such as anti-HIV, anti-tuberculosis,^29^ antitumor,^30^ anti-cancer, anti-anaphylactic,^31^ and anticoagulant^32^ activities.

By the way, oxygen analogues of polycyclic aromatic hydrocarbons^33^ represent an important class of molecules because the incorporation of oxygen atoms into an aromatic hydrocarbon system can modulate its pharmacological activity as well as electrical or optical properties.^34^ Therefore, the synthesis of these class of chromene compounds has fascinated much noticed in organic synthesis and many researchers have reported synthesis of these important compounds over the past few years.^35–49^

Ketene dithioacetals are important precursors in organic synthesis of a variety of heterocyclic compounds and they have received increasing attention recently.^50–54^ As part of our continuing research to be develop novel methods for multi-component synthesis of new biologically active heterocyclic compounds from dithioacetals and because of the importance of using RSM in optimization of reaction conditions, herein, we report an efficient synthesis of 4,4′-(1,4-phenylene)bis(2-(alkylamino)-3-nitro-4*H*-benzo[*g*]chromene-5,10-dione) and 4,4′-(1,4-phenylene)bis(2-(alkylamino)-3-nitropyrano[3,2-*c*]chromen-5(4*H*)-one) derivatives *via* one-pot, multi-component reaction of various amines, 1,1-bis(methylthio)-2-nitroethene (nitro ketene dithioacetal), terephthalaldehyde or isophthalaldehyde, and 2-hydroxy-1,4-naphthoquinone or 4-hydroxycoumarin in EtOH/H_2_O (85 : 15) at 89 °C without any catalyst. Central composite design (CCD) one of the most popular RSM^55^ is selected as the experimental design method for optimizing reaction conditions.

## Result and discussion

**Scheme 1:**
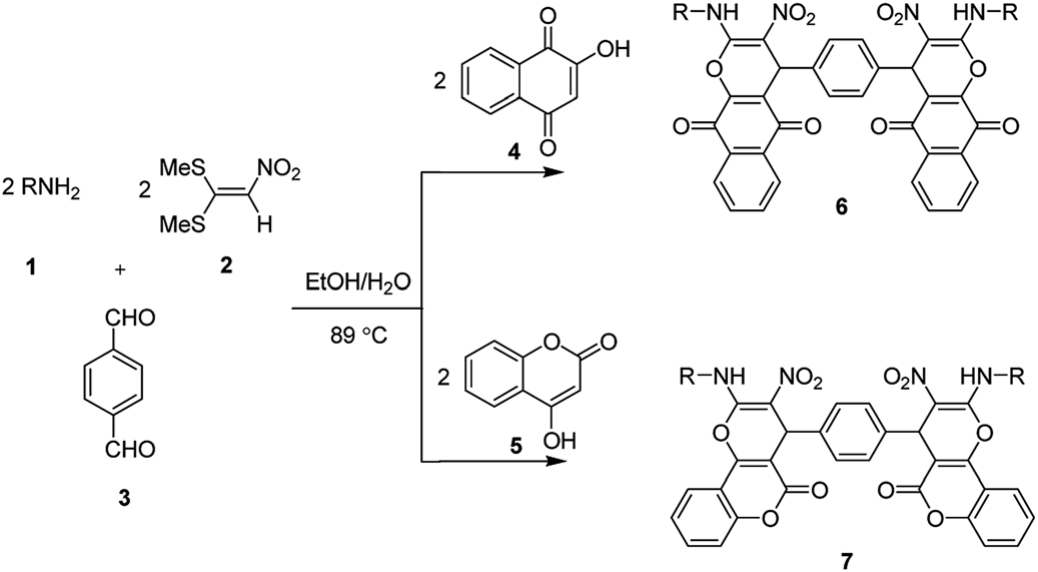
Synthetic scheme for the products 6 and 7.

In the current study, we introduce an efficient one-pot, multi-component reaction of various amines 1, 1,1-bis(methylthio)-2-nitroethene 2, terephthalaldehyde or isophthalaldehyde 3, and 2-hydroxy-1,4-naphthoquinone 4 or 4-hydroxycoumarin 5 for the synthesis of 4,4′-(1,4-phenylene)bis(2-(alkylamino)-3-nitro-4*H*-benzo[*g*]chromene-5,10-dione) 6 and 4,4′-(1,4-phenylene)bis(2-(alkylamino)-3-nitropyrano[3,2-*c*]chromen-5(4*H*)-one) 7 derivatives in EtOH/H_2_O (85 : 15) as solvent at 89 °C without any catalyst (Scheme 1).

**Scheme 2:**
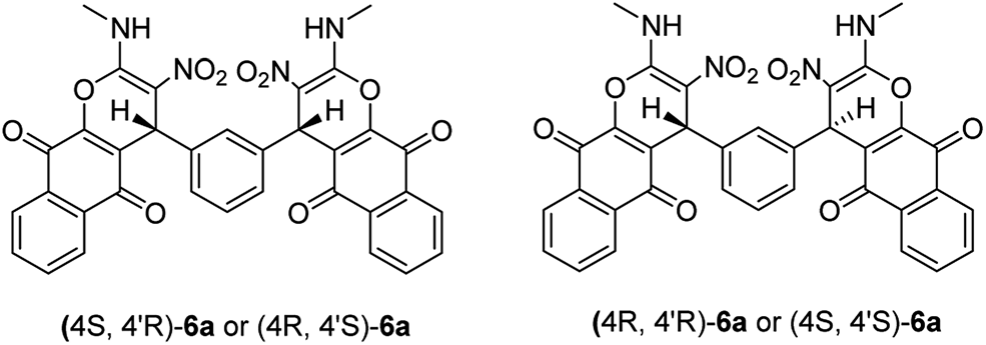
The two diastereoisomers of 6a.

Response surface methodology (RSM) using central composite design (CCD) with five replicates at the center point was applied to optimize the reaction conditions. We used the reaction of *N*-methyl-1-(methylthio)-2-nitroethenamine (2 mmol), terephthalaldehyde (1 mmol), and 2-hydroxy-1,4-naphthoquinone (2 mmol) for the preparation of compound 6b as a model. The two independent variables (temperature (*A*) and water content of aqueous ethanol (*B*)), levels (Table 1), and the results obtained after running the experiments are represented in Table 2. The two responses were analyzed: yield of product (*R*_1_) and reaction time (*R*_2_).

**Table 1:** Selected variables and levels used in central composite design

Variables	Code	Units	Levels		
			−1	0	+1
Reaction temperature	*A*	°C	25	62.5	100
Water content of aqueous ethanol	*B*	%	0	50	100

**Table 2:** Process variables and experimental data for two factors, three levels response surface design^a^

Run	*A* (°C)	*B* (%)	*R*_1_ (%)	*R*_2_ (h)
1	36	85	0	24
2	100	50	64	0.25
3	62	100	5	24
4	62	50	20	7
5	62	50	26	7
6	62	50	16	7
7	62	50	22	7
8	62	0	47	2
9	36	15	27	24
10	89	15	84	0.5
11	62	50	30	7
12	89	85	5	24
13	25	50	0	24

a *A* = temperature (°C), *B* = water content of aqueous ethanol (%), *R*_1_ = yield of reaction, *R*_2_ = reaction time.

Optimum conditions with respect to yield, purity of product and reaction time were as follow: temperature 89 °C, water content of aqueous ethanol 15%. Verification experiments, carried out at the predicted conditions showed values reasonably close to those predicted and further confirmed the adequacy of predicted models.

On the basis of the CCD, the cubic model relationship between the experimental yield (*R*_1_) and the process variables (temperature (*A*) and water content of aqueous ethanol (*B*)) and the quadratic model between the reaction time (*R*_2_) and the process variables, in coded units are obtained from eqn (1) and (2) respectively.

1 *R*_1_ = 22.80 + 22.63*A* − 14.85*B* − 13.00*AB* + 4.60*A*^2^ + 1.60*B*^2^ − 11.65*A*^2^*B* − 7.13*AB*^2^

2 *R*_2_ = 7.00 − 7.14*A* + 6.83*B* + 5.88*AB* + 3.95*A*^2^ + 4.39*B*^2^

where *R*_1_ and *R*_2_ represent the experimental yield and reaction time respectively, then *A* (temperature) and *B* (water content of aqueous ethanol) are the coded variables in the reaction.

**Fig. 1:**
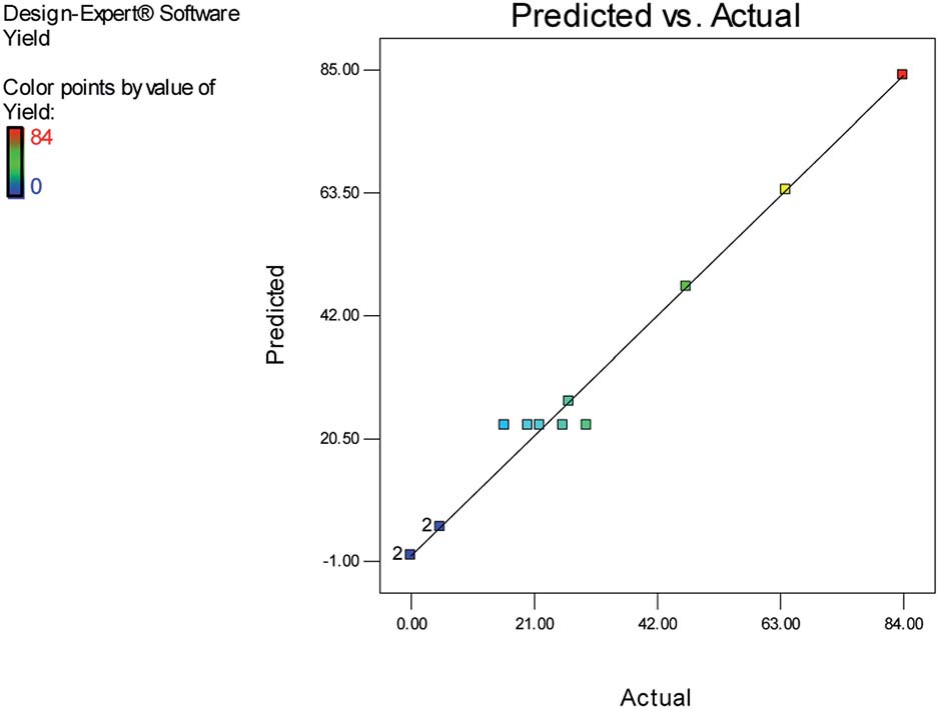
Linear correlation between the actual and predicted yield.

**Fig. 2:**
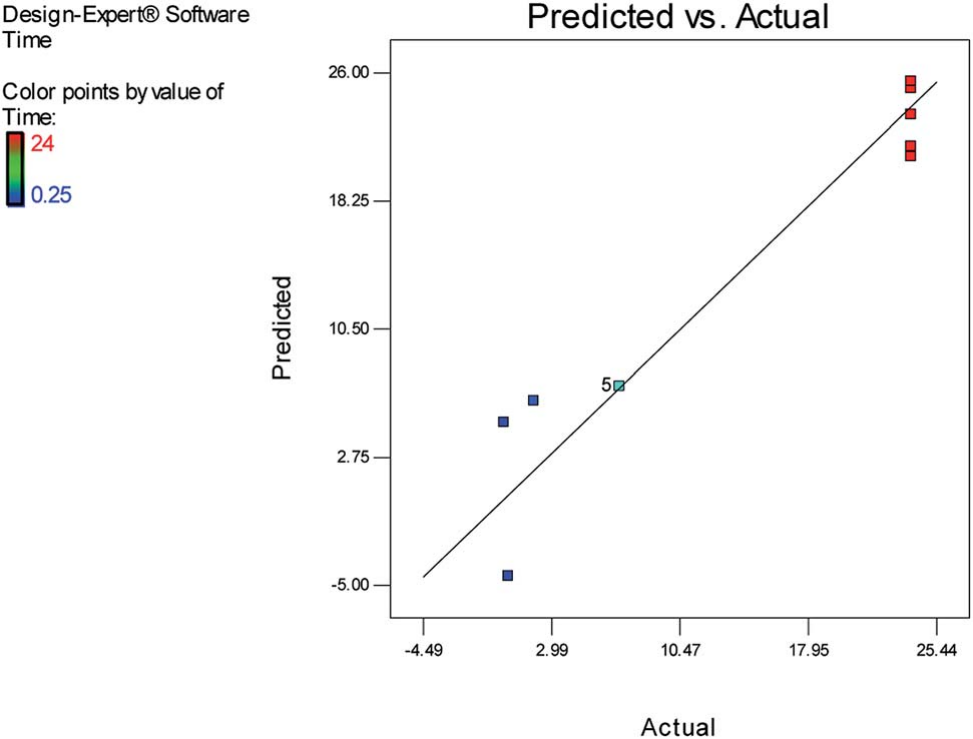
Linear correlation between the actual and predicted time.

Fig. 1 and 2 show the linear correlation between the actual and predicted yield and time, respectively. According to the figures, the predicted yield is consistent with the experimental yield and the model can be used to predict the yield of product successfully.

The ANOVA for response surface cubic and quadratic models are reported in Tables 3 and 4. According to the Table 3, the model *F*-value of 46.05 implies the cubic model is significant. There is only a 0.03% chance that a “Model *F*-Value” this large could occur due to noise. Values of “Prob > *F*” less than 0.0500 indicate model terms are significant. In this case *A*, *B*, *AB*, *A*^2^*B* are significant model terms. The “Lack of Fit *F*-value” of 0.00 implies the Lack of Fit is not significant relative to the pure error. There is a 100.00% chance that a “Lack of Fit *F*-value” this large could occur due to noise. Non-significant Lack of Fit is good and we want the model to fit. The “Pred. *R*-Squared” of 0.9761 is in reasonable agreement with the “Adj. *R*-Squared” of 0.9633. “Adeq. Precision” measures the signal to noise ratio. A ratio greater than 4 is desirable. Our ratio of 22.155 indicates an adequate signal. According to the Table 4, the model *F*-value of 19.38 implies the quadratic model is significant. There is only a 0.06% chance that a “Model *F*-Value” this large could occur due to noise. The “Pred. *R*-Squared” of 0.5210 is not as close to the “Adj. *R*-Squared” of 0.8845 as one might normally expect. This may indicate a large block effect. Also “Adeq. Precision” measures the signal to noise ratio. A ratio greater than 4 is desirable. Our ratio of 12.884 indicates an adequate signal.

**Table 3:** ANOVA for response surface cubic model

Source	Sum of Square	Degree of freedom	Mean square	*F* value	*p*-value Prob > *F*
Model	7530.28	7	1075.75	46.05	0.0003 significant
*A*-temperature	2048.00	1	2048.00	87.67	0.0002
*B*-percentage of water in ethanol	882.00	1	882.00	37.76	0.0017
*AB*	676.00	1	676.00	28.94	0.0030
*A* ^2^	147.20	1	147.20	6.30	0.0538
*B* ^2^	17.81	1	17.81	0.76	0.4225
*A* ^2^ *B*	271.48	1	271.48	11.62	0.0191
*AB* ^2^	101.60	1	101.60	4.35	0.0914
*A* ^3^	0.000	0			
*B* ^3^	0.000	0			
Residual	116.80	5	23.36		
Lack of fit	0.000	1	0.000	0.000	1.0000 not significant
Pure error	116.80	4	29.20		
Correlation total	7647.08	12			

**Table 4:** ANOVA for response surface quadratic model

Source	Sum of square	Degree of freedom	Mean square	*F* value	*p*-value
Model	1133.23	5	226.65	19.38	0.0006 significant
*A*-temperature	407.37	1	407.37	34.84	0.0006
*B*-percentage of water in ethanol	372.82	1	372.82	31.89	0.0008
*AB*	138.06	1	138.06	11.81	0.0109
*A* ^2^	108.71	1	108.71	9.30	0.0186
*B* ^2^	134.10	1	134.10	11.47	0.0117
Residual	81.85	7	11.69		
Lack of fit	81.85	3	27.26		
Pure error	0.000	4	0.000		
Correlation total	1215.08	12			

**Fig. 3:**
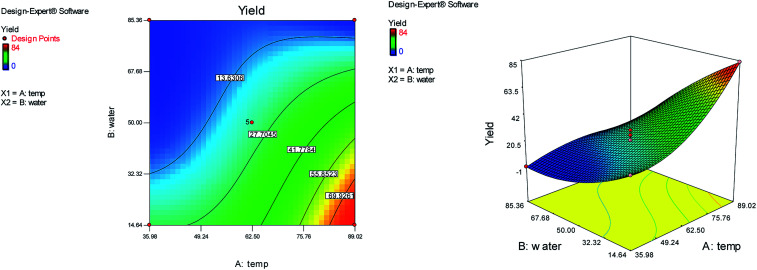
The effects of temperature and water content of aqueous ethanol on the total reaction yield.

**Fig. 4:**
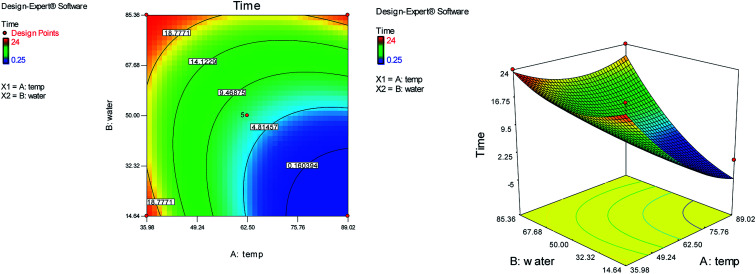
The effects of temperature and the water content of aqueous ethanol on the total reaction time.

The effects of temperature and water content of aqueous ethanol on the total reaction yield, and the total reaction time are shown in Fig. 3 and 4, respectively. The total yield varied from 0% to 84%. As temperature increased, the yield increased and the reaction time decreased. The changes in yield *versus* the water content of aqueous ethanol is minor compared to that of temperature. As the water content of aqueous ethanol decreased, the yield increased and the reaction time decreased. The highest yield (84%) was obtained at 89 °C and at 15% water. The lowest yield (0%) were at 36 °C, 85% water, and at 25 °C, 50% water.

**Scheme 3:**
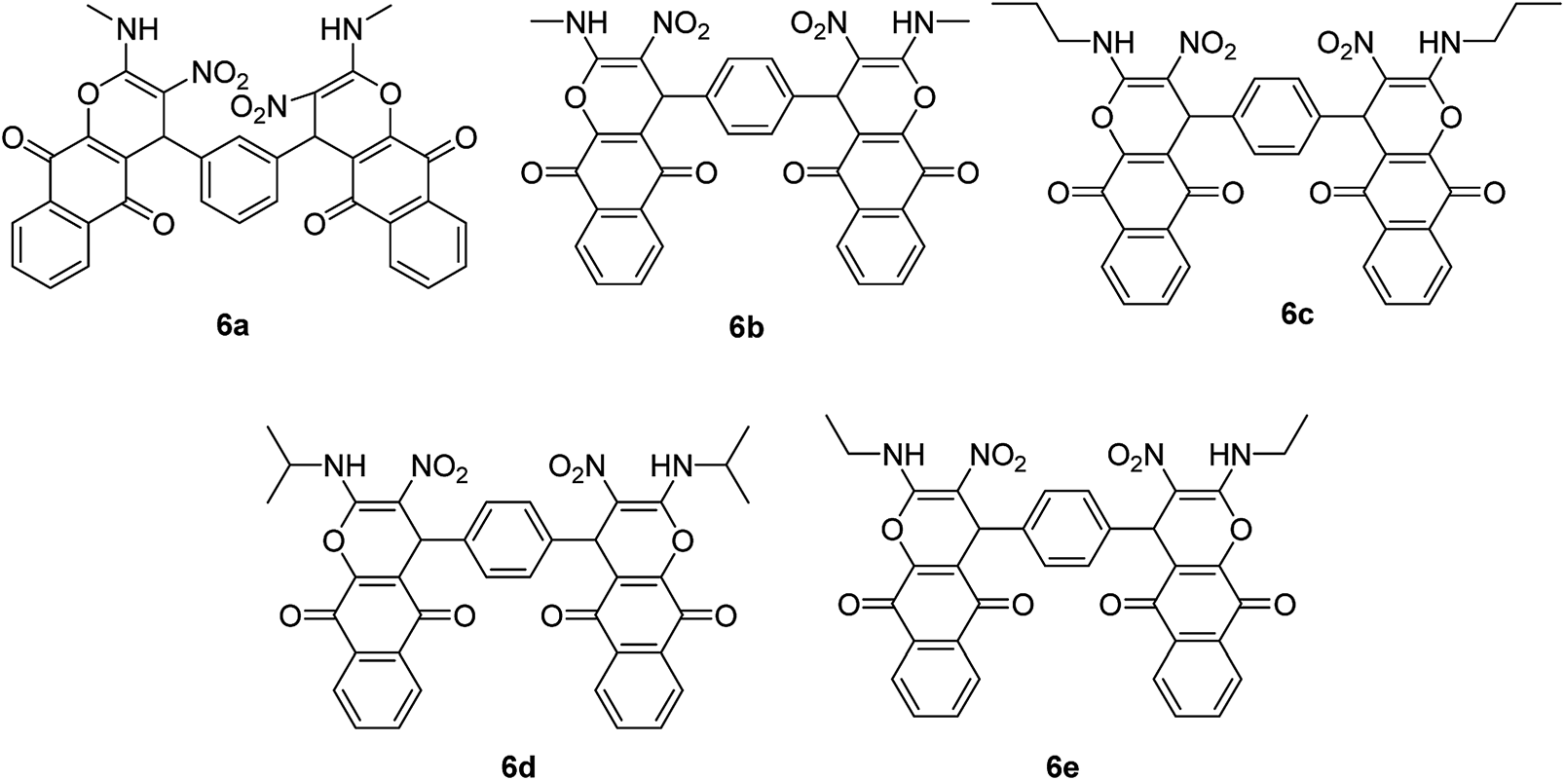
Products 6a–e.

**Scheme 4:**
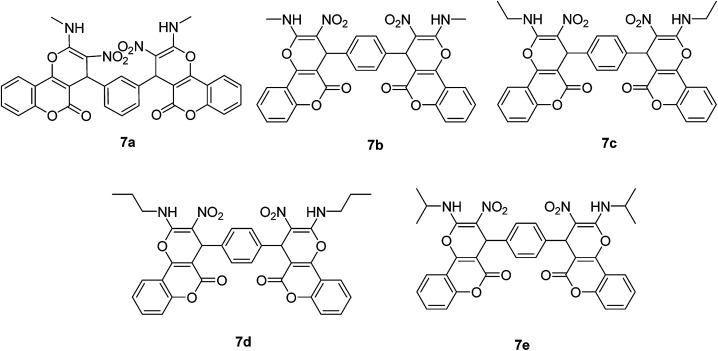
Products 7a–e.

After obtaining the optimized reaction conditions, we investigated the scope of this reaction by varying the structure of the amines 1, phthalaldehydes 3, and CH acids (4 and 5). The reaction proceeds cleanly under the same reaction conditions to afford corresponding fused heterocyclic derivatives 6 and 7 (Schemes 3 and 4) in 75–91% yields. The results are shown in Table 5. The reaction did not work, when the reaction was performed using *ortho*-phthalaldehyde, and benzylamine. The steric hindrance makes it less reactive toward reaction.

**Table 5:** One-pot, multi-component synthesis of bis(benzo[*g*]chromene) (6a–e) and bis(pyrano[3,2-*c*]chromene) derivatives (7a–e)

Entry	RNH_2_^a^	Phthalaldehyde^a^	Product	Time (min)	Yield (%)
1	Methylamine	Isophthalaldehyde	6a	25	86
2	Methylamine	Terephthalaldehyde	6b	30	84
3	Propylamine	Terephthalaldehyde	6c	40	81
4	Isopropylamine	Terephthalaldehyde	6d	35	79
5	Ethylamine	Terephthalaldehyde	6e	45	75
6	Methylamine	Isophthalaldehyde	7a	15	87
7	Methylamine	Terephthalaldehyde	7b	15	91
8	Ethylamine	Terephthalaldehyde	7c	30	82
9	Propylamine	Terephthalaldehyde	7d	20	89
10	Isopropylamine	Terephthalaldehyde	7e	25	85
11	Benzylamine	Terephthalaldehyde	n.r.	—	—
12	Methylamine	Orthophthalaldehyde	n.r.	—	—

a Various amines (2 mmol), 1,1-bis(methylthio)-2-nitroethene (2 mmol), phthalaldehyde (1 mmol), 2-hydroxy-1,4-naphthoquinone (2 mmol) and 4-hydroxycumarin (2 mmol) were used. The reactions were run in EtOH/H_2_O (85 : 15) at 89 °C, without any catalyst.

Structural elucidation of the newly synthesized compounds was accomplished using their IR, mass, ^1^H NMR, and ^13^C NMR spectroscopy (see the ESI†). Compounds 6 and 7 have two stereogenic centers, and therefore two diastereoisomers are expected. For examples the two diastereoisomers of 6a is shown in Scheme 2. The ^1^H and ^13^C NMR spectra of the products are consistant with the presence of two diastereoisomers. We were not able to separate compounds 6 and 7 in pure form. However, their NMR data can be extracted from the mixture of the two diastereoisomers in a nearly 50 : 50 ratio (based on integration of methine protons). The ^1^H and ^13^C NMR spectra of two diastereoisomers are similar except for the alkyl amine and methine groups. Also we did not obtain the good NMR spectra of these samples because of its insolubility in any solvent (all products are very insoluble compounds).

For examples the IR spectrum of 6b indicated absorption bands due to the NH stretching (3613 cm^−1^) as well as bands at 1667, 1467 and 1363, 1245, and 1192 cm^−1^ due to the C=O, NO_2_, C–N, and C–O groups. The ^1^H NMR spectrum of 6b showed multiplets for four CH_3_ groups (*δ* 3.15–3.23 ppm), two singlets for four CH groups (*δ* 5.23 and 5.28 ppm), multiplets for the aromatic region (*δ* 7.01–8.04 ppm), and multiplets for four NH groups (*δ* 10.23–10.35 ppm). The ^1^H-decoupled ^13^C NMR spectrum of 6b showed three signals for CH_3_ and CH groups (*δ* 29.9, 37.4, 38.0 ppm), and signals at 108.5, 177.6, 182.8, 183.5, and 184.7 ppm, which were assigned C–NO_2_ and C=O groups, respectively. The mass spectrum of this structure was in accordance with the proposed structure.

**Scheme 5:**
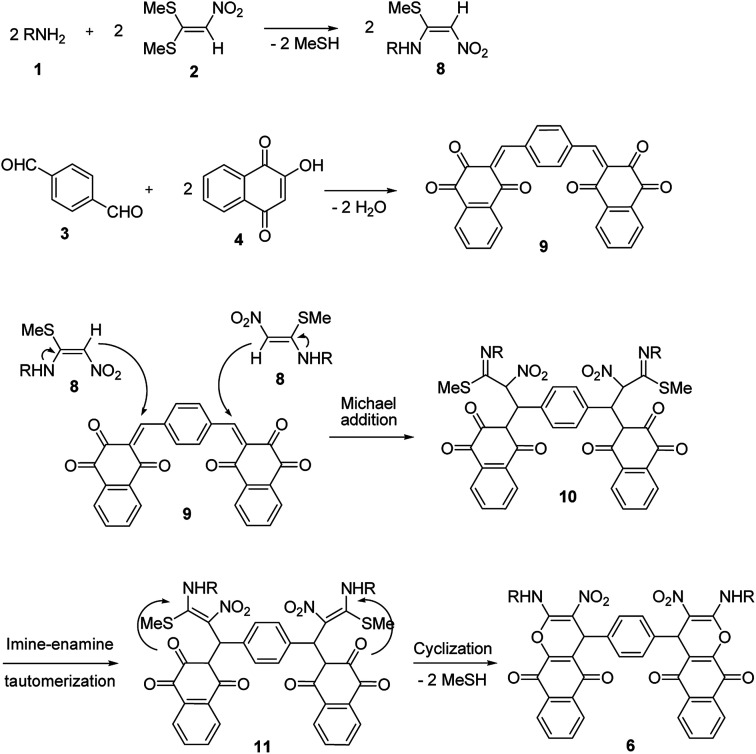
Proposed mechanism for the synthesis of product 6.

**Scheme 6:**
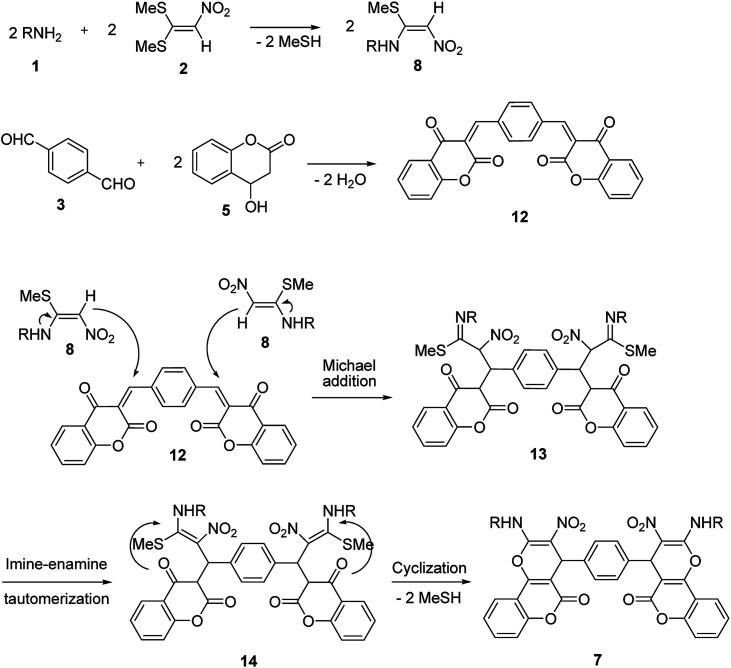
Plausible mechanism for the formation of product 7.

A proposed mechanism for the synthesis of 6 and 7 is shown in Schemes 5 and 6. Initially, the Knoevenagel condensation between terephthalaldehyde 3 (1 mmol) and 2-hydroxy-1,4-naphthoquinone 4 (or 4-hydroxycumarin 5) (2 mmol) affords 9 (or 12) which undergoes Michael addition with *N*-alkyl-1-(methylthio)-2-nitroethenamine 8 (2 mmol) derived from the addition of alkylamine 1 (2 mmol) to 1,1-bis(methylthio)-2-nitroethene 2 (2 mmol), to give 10 (or 13). Thus the intermediate 10 (or 13) undergoes imine-enamine tautomerisation to form 11 (or 14) followed by *O*-cyclization to form 6 (or 7) *via* the elimination of MeSH.

## Experimental

### General

The various amines, 1,1-bis(methylthio)-2-nitroethene, terephthalaldehyde, isophthalaldehyde, 2-hydroxy-1,4-naphthoquinone, 4-hydroxycumarin, and solvents were purchased from Sigma-Aldrich chemical company and were used as received without further purification. Melting points were determined with an electrothermal 9100 apparatus. Infrared (IR) spectra were recorded on a Bruker Tensor 27 spectrometer. Nuclear magnetic resonance (NMR) spectra were obtained on a Bruker DRX-300 Avance instrument (300 MHz for ^1^H and 75.4 and 62.8 MHz for ^13^C) with DMSO as solvent. Chemical shifts are expressed in parts per million (ppm), and coupling constant (*J*) are reported in hertz (Hz). Mass spectra were recorded with an Agilent 5975C VL MSD with Triple-Axis Detector operating at an ionization potential of 70 eV.

### General procedure for the synthesis of product 6 and 7

A mixture of amine (2 mmol), 1,1-bis(methylthio)-2-nitroethene (2 mmol, 0.330 g) and 10 mL EtOH/H_2_O (85 : 15) in a 50 mL flask was stirred for 6 hours at 89 °C. After completion of the reaction (monitored by TLC, ethyl acetate/*n*-hexane, 1 : 1), phthalaldehyde (1 mmol), 2-hydroxy-1,4-naphthoquinone (2 mmol, 0.348 g) or 4-hydroxycumarin (2 mmol, 0.324 g) were added to the reaction mixture, and it was stirred at 89 °C for the time given in Table 5. Then, the reaction mixture was filtered to give the crude product. The solid was washed with EtOH to give pure product 6 or 7 in good to high yield.

#### 4,4′-(1,3-phenylene)bis(2-(methylamino)-3-nitro-4*H*-benzo[*g*]chromene-5,10-dione) (6a)

Orange solid: mp: 302–305 °C, yield: 0.556 g (86%). ^1^H NMR (300 MHz, DMSO-*d*_6_): *δ* 3.19 (6H, s, 2CH_3_), 3.21 (6H, s, 2CH_3_), 5.24 (2H, s, 2CH), 5.27 (2H, s, 2CH), 7.13–8.07 (24H, m, ArH), 10.28 (4H, m, 4NH). ^13^C NMR (75 MHz, DMSO-*d*_6_): *δ* 29.0 (4NCH_3_), 36.6 (2CH), 36.8 (2CH), 107.2 (2C–NO_2_), 107.4 (2C–NO_2_), 123.5, 124.5, 125.0, 125.1, 126.5, 127.2, 129.5, 130.7, 131.2, 134.9, 135.1, 141.3, 147.5, 147.8, 157.4, 157.5, 176.5 (4C=O), 182.2 (2C=O), 182.3 (2C=O).

#### 4,4′-(1,4-phenylene)bis(2-(methylamino)-3-nitro-4*H*-benzo[*g*]chromene-5,10-dione) (6b)

Orange solid: mp: 249–251 °C, yield: 0.543 g (84%). IR (KBr) (*ν*_max_/cm^−1^): 3613, 1667, 1467, 1363, 1245, 1192. MS (EI, 70 eV): *m*/*z* (%) = 390 (57), 361 (10), 333 (16), 84 (66), 66 (100). ^1^H NMR (300 MHz, DMSO-*d*_6_): *δ* 3.15–3.23 (12H, m, 4CH_3_), 5.23 (2H, s, 2CH), 5.28 (2H, s, 2CH), 7.01–8.04 (24H, m, ArH), 10.23–10.35 (4H, m, 4NH). ^13^C NMR (62 MHz, DMSO-*d*_6_): *δ* 29.9 (4NCH_3_), 37.4 (2CH), 38.1 (2CH), 108.5 (2C–NO_2_), 124.2, 126.9, 127.5, 129.0, 129.4, 129.8, 131.4, 131.9, 132.4, 133.7, 134.3, 135.9, 139.1, 141.3, 148.9, 158.7, 177.6 (2C=O), 182.8 (2C=O), 183.5 (2C=O), 184.7 (2C=O).

#### 4,4′-(1,4-phenylene)bis(3-nitro-2-(propylamino)-4*H*-benzo[*g*]chromene-5,10-dione) (6c)

Orange solid: mp: 264–266 °C, yield: 0.569 g (81%). IR (KBr) (*ν*_max_/cm^−1^): 3457, 1678, 1522, 1380, 1233, 1146. MS (EI, 70 eV): *m*/*z* (%) = 390 (100), 361 (12), 333 (24), 276 (25), 246 (81), 174 (20), 143 (37), 104 (58), 68 (55). ^1^H NMR (300 MHz, DMSO-*d*_6_): *δ* 0.80 (12H, t, 4CH_3_), 1.50–1.61 (8H, m, 4CH_2_), 2.75–2.80 (8H, m, 4CH_2_), 5.44 (4H, s, 4CH), 7.53–7.86 (24H, m, Ar), 8.80 (2H, br s, 2NH), 9.94 (2H, br s, 2NH).

#### 4,4′-(1,4-phenylene)bis(2-(isopropylamino)-3-nitro-4*H*-benzo[*g*]chromene-5,10-dione) (6d)

Orange solid: mp: 281–283 °C, yield: 0.555 g (79%). ^1^H NMR (300 MHz, DMSO-*d*_6_): *δ* 1.10–1.23 (24H, m, 8CH_3_), 4.30 (4H, m, 4CH), 5.51 (4H, s, 4CH), 7.38–7.87 (24H, m, Ar), 8.80 (2H, br s, 2NH), 9.94 (2H, br s, 2NH).

#### 4,4′-(1,4-phenylene)bis(2-(ethylamino)-3-nitro-4*H*-benzo[*g*]chromene-5,10-dione) (6e)

Orange solid: mp: 279–281 °C, yield: 0.505 g (75%). ^1^H NMR (300 MHz, DMSO-*d*_6_): *δ* 1.12 (12H, t, ^3^*J*_HH_ = 7.5 Hz, 4CH_3_), 2.80–2.90 (8H, m, 4CH_2_), 5.45 (4H, s, 4CH), 7.50–8.87 (24H, m, Ar), 8.80 (2H, br s, 2NH), 9.65 (2H, br s, 2NH). ^13^C NMR (62 MHz, DMSO-*d*_6_): *δ* 12.4 (2CH_3_), 13.9 (2CH_3_), 36.2 (2CH_2_), 36,4 (2CH_2_), 37.0 (2CH), 38.0 (2CH), 111.0 (4C–NO_2_), 126.5, 127.1, 128.3, 128.7, 129.1, 130.7, 131.7, 132.4, 133.2, 134.5, 135.1, 136.1, 177.0 (2C=O), 183.7 (2C=O), 184.4 (2C=O), 185.0 (2C=O).

#### 4,4′-(1,3-phenylene)bis(2-(methylamino)-3-nitropyrano[3,2-*c*]chromen-5(4*H*)-one) (7a)

White solid: mp: 312–314 °C, yield: 0.541 g (87%). IR (KBr) (*ν*_max_/cm^−1^): 3610, 1729, 1674, 1458, 1364, 1266, 1160. MS (EI, 70 eV): *m*/*z* (%) = 256 (6), 200 (12), 136 (11), 96 (13), 81 (46), 69 (100). ^1^H NMR (300 MHz, DMSO-*d*_6_): *δ* 3.16–3.29 (12H, m, 4CH_3_), 5.03 (4H, s, 4CH), 7.13–8.02 (24H, m, Ar), 10.25–10.40 (4H, m, 4NH). ^13^C NMR (62 MHz, DMSO-*d*_6_): *δ* 30.1 (4NHCH_3_), 38.4 (4CH), 108.2, 109.0, 113.9, 118.0, 124.1, 126.5, 128.2, 129.3, 129.5, 134.6, 142.6, 153.4, 158.3, 160.5.

#### 4,4′-(1,4-phenylene)bis(2-(methylamino)-3-nitropyrano[3,2-*c*]chromen-5(4*H*)-one) (7b)

White solid: mp: 325–327 °C, yield: 0.566 g (91%). ^1^H NMR (300 MHz, DMSO-*d*_6_): *δ* 3.25–3.32 (12H, m, 4CH_3_), 5.02 (4H, s, 4CH), 7.19–8.02 (24H, m, Ar), 10.30–10.35 (4H, m, 4NH). ^13^C NMR (62 MHz, DMSO-*d*_6_): *δ* 30.1 (4NCH_3_), 38.3 (4CH), 109.2, 118.0, 124.2, 126.4, 129.5, 134.5, 141.4, 153.5, 158.3, 160.5.

#### 4,4′-(1,4-phenylene)bis(2-(ethylamino)-3-nitropyrano[3,2-*c*]chromen-5(4*H*)-one) (7c)

White solid: mp: 290–292 °C, yield: 0.533 g (82%). MS (EI, 70 eV): *m*/*z* (%) = 256 (7), 137 (15), 97 (38), 81 (73), 69 (100). ^1^H NMR (300 MHz, DMSO-*d*_6_): *δ* 1.30 (12H, t, 4CH_3_), 3.70–3.85 (8H, m, 4CH_2_NH), 5.02 (4H, s, 4CH), 7.06–7.93 (24H, m, Ar), 10.43 (4H, s, 4NH). ^13^C NMR (62 MHz, DMSO-*d*_6_): *δ* 16.5 (4CH_3_), 38.3 (4CH), 38.5 (4CH_2_), 104.6, 108.1, 109.1, 114.1, 116.8, 118.1, 121.3, 124.0, 125.6, 126.4, 127.9, 129.0, 129.5, 130.5, 132.2, 134.5, 139.0, 141.4, 153.5, 157.8, 160.6, 179.2.

#### 4,4′-(1,4-phenylene)bis(3-nitro-2-propylaminopyrano[3,2-*c*]chromen-5(4*H*)-one) (7d)

White solid: mp: 297–299 °C, yield: 0.603 g (89%). H NMR (300 MHz, DMSO-*d*_6_): *δ* 0.90 (12H, t, 4CH_3_), 1.70–1.80 (8H, m, 4CH_2_), 3.65–3.80 (8H, m, 4CH_2_NH), 5.03 (4H, s, 4CH), 7.05–7.98 (24H, m, Ar), 10.44 (4H, s, 4NH). ^13^C NMR (62 MHz, DMSO-*d*_6_): *δ* 12.6 (4CH_3_), 24.2 (4CH_2_), 37.3 (4CH), 44.8 (4CH_2_NH), 104.6, 108.1, 109.1, 114.1, 116.8, 118.1, 121.3, 123.9, 125.6, 126.5, 127.9, 129.0, 129.5, 132.2, 134.5, 141.4, 153.4, 157.9, 160.6, 169.0.

#### 4,4′-(1,4-phenylene)bis(2-(isopropylamino)-3-nitropyrano[3,2-*c*]chromen-5(4*H*)-one) (7e)

White solid: mp: 313–315 °C, yield: 0.576 g (85%). H NMR (300 MHz, DMSO-*d*_6_): *δ* 1.30–1.40 (24H, d, 8CH_3_), 4.45–4.60 (4H, m, 4CHNH), 5.02 (4H, s, 4CH), 7.19–7.95 (24H, m, Ar), 10.10–10.25 (4H, m, 4NH). ^13^C NMR (62 MHz, DMSO-*d*_6_): *δ* 23.8 (8CH_3_), 38.2 (4CH), 46.4 (4CHNH), 104.6, 114.1, 116.8, 118.1, 121.5, 124.2, 125.6, 127.9, 129.0, 129.6, 130.7, 132.2, 134.6, 153.4, 153.9, 157.4, 160.6, 169.0.

## Conclusion

In summary, a simple and catalyst free method for the synthesis of 4,4′-(1,4-phenylene)bis(2-(alkylamino)-3-nitro-4*H*-benzo[*g*]chromene-5,10-dione) and 4,4′-(1,4-phenylene)bis(2-(alkylamino)-3-nitropyrano[3,2-*c*]chromen-5(4*H*)-one) derivatives was reported for the first time. These compounds are obtained from a one-pot, multi-component reaction between various amines, 1,1-bis(methylthio)-2-nitroethene, terephthalaldehyde or isophthalaldehyde, and 2-hydroxy-1,4-naphthoquinone or 4-hydroxycoumarin in EtOH/H_2_O (85 : 15) at 89 °C. After optimization of reaction conditions by response surface methodology (RSM) all the products could be obtained in good yields (from 75% to 91%). The key advantages of this new synthetic route are atom economy, environmental friendliness, simple operational process and the non-chromatographic purification of products. This study has shown that the experimental results are in good agreement with the predicted values, and the model successfully can be used to predict the synthesis of such derivatives. Further studies on the synthetic application and physical properties examination are currently ongoing.

## Conflicts of interest

The authors declare no competing financial interest.

## Supplementary Material

RA-009-C9RA07809F-s001
