# Supplementary material for: Rapid and catalyst free synthesis of new bis(benzo[g]chromene) and bis(pyrano[3,2-c]chromene) derivatives and optimization of reaction conditions using response surface methodology
Source: RSC Adv. 2019 Dec 2;9(67):39466–74. doi: 10.1039/c9ra07809f (PMC9076105; doi:10.1039/c9ra07809f)
Supplement: RA-009-C9RA07809F-s001 [file RA-009-C9RA07809F-s001.pdf]

Electronic Supplementary Material (ESI) for RSC Advances.
This journal is © The Royal Society of Chemistry 2019

Supporting Information

Rapid and catalyst free synthesis of new bis(benzo[g]chromene) and bis(pyrano[3,2-c]chromene) derivatives and optimization of reaction conditions using response surface methodology

Fahimeh Sadat Hosseini, Mohammad Bayat*[a], Milad Afsharnezhad

[a] *Chemistry Department, Imam Khomeini International University, Qazvin, Iran*

E-mail: bayat_mo@yahoo.com; m.bayat@sci.ikiu.ac.ir

The Table of Contents

Title	Page
Title, author's name, address and table of contents	1
Experimental section	2
**Figure 1**. Structure of all products **6a-e** and **7a-e**	3
^{1}H and ^{13}C NMR spectrums of **6a**	4-5
^{1}H and ^{13}C NMR and IR and Mass spectrums of **6b**	6-9
^{1}H NMR and IR and Mass spectrums of **6c**	10-12
^{1}H NMR spectrum of **6d**	13
^{1}H and ^{13}C NMR spectrums of **6e**	14-15
^{1}H and ^{13}C NMR and IR and Mass spectrums of **7a**	16-19
^{1}H and ^{13}C NMR spectrums of **7b**	20-21
^{1}H and ^{13}C NMR and Mass spectrums of **7c**	22-24
^{1}H and ^{13}C NMR spectrums of **7d**	25-26
^{1}H and ^{13}C NMR spectrums of **7e**	27-28

Experimental

General

The various amines, 1,1-bis(methylthio)-2-nitroethene, terephthalaldehyde, Isophthalaldehyde, 2-hdroxy-1,4-naphthoquinone, 4-hydroxycumarin, and solvents were purchased from Sigma-Aldrich chemical company and were used as received without further purification. Melting points were determined with an electrothermal 9100 apparatus. Infrared (IR) spectra were recorded on a Bruker Tensor 27 spectrometer. Nuclear magnetic resonance (NMR) spectra were obtained on a Bruker DRX-300 Avance instrument (300 MHz for ^{1}H and 75.4 and 62.8 MHz for ^{13}C) with DMSO as solvent. Chemical shifts are expressed in parts per million (ppm), and coupling constant (*J*) are reported in hertz (Hz). Mass spectra were recorded with an Agilent 5975C VL MSD with Triple-Axis Detector operating at an ionization potential of 70 eV.

General procedure for the synthesis of product 6 and 7

A mixture of amine (2 mmol), 1,1-bis(methylthio)-2-nitroethene (2 mmol, 0.330 g) and 10 mL EtOH/H_2O (85:15) in a 50 mL flask was stirred for 6 hours at 89 ˚C. After completion of the reaction (monitored by TLC, ethyl acetate/n-hexane, 1:1), phthaldialdehyde (1 mmol), 2-hydroxy-1,4-naphthoquinone (2 mmol, 0.348 g) or 4-hydroxycumarin (2 mmol, 0.324 g) were added to the reaction mixture, and it was stirred at 89 ˚C for the time given in Table 5. Then, the reaction mixture was filtered to give the crude product. The solid was washed with EtOH to give pure product **6** or **7** in good to high yield.

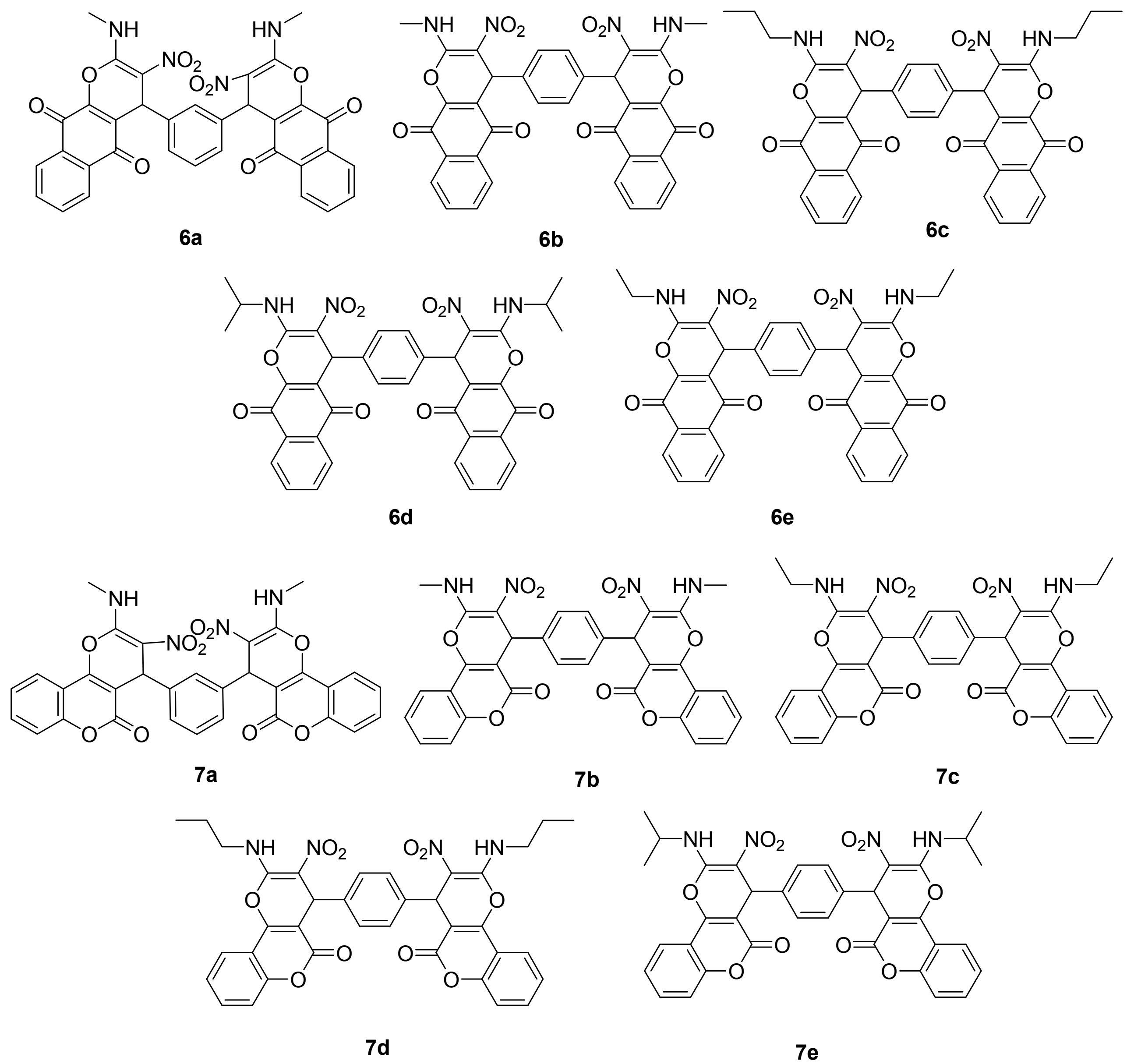

Figure 1. Structure of all products **6a-e** and **7a-e.**

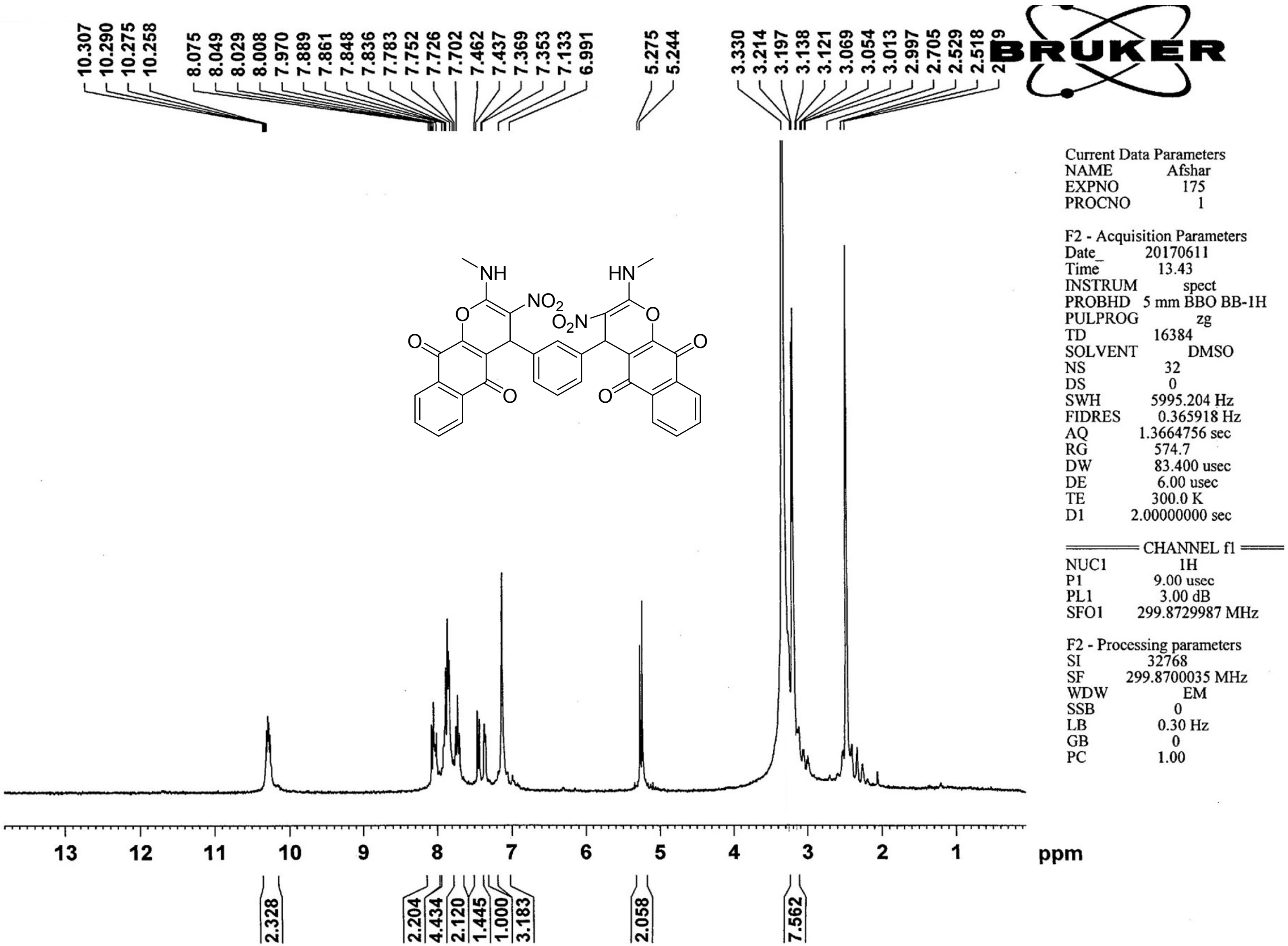
10.307
10.290
10.275
10.258
8.075
8.049
8.029
8.008
7.970
7.889
7.861
7.848
7.836
7.783
7.752
7.726
7.702
7.462
7.437
7.369
7.353
7.133
6.991
5.275
5.244
3.330
3.214
3.197
3.138
3.121
3.069
3.054
3.013
2.997
2.705
2.529
2.518
BRUKER
NH
HN
NO2
O2N
O
Current Data Parameters
NAME Afshar
EXPNO 175
PROCNO 1
F2 - Acquisition Parameters
Date_ 20170611
Time 13.43
INSTRUM spect
PROBHD 5 mm BBO BB-1H
PULPROG zg
TD 16384
SOLVENT DMSO
NS 32
DS 0
SWH 5995.204 Hz
FIDRES 0.365918 Hz
AQ 1.3664756 sec
RG 574.7
DW 83.400 usec
DE 6.00 usec
TE 300.0 K
D1 2.00000000 sec
CHANNEL f1
NUC1 1H
P1 9.00 usec
PL1 3.00 dB
SFO1 299.8729987 MHz
F2 - Processing parameters
SI 32768
SF 299.8700035 MHz
WDW EM
SSB 0
LB 0.30 Hz
GB 0
PC 1.00
13
12
11
10
9
8
7
6
5
4
3
2
1
ppm
2.328
2.204
4.434
2.120
1.445
1.000
3.183
2.058
7.562

^{1}H NMR of 6a

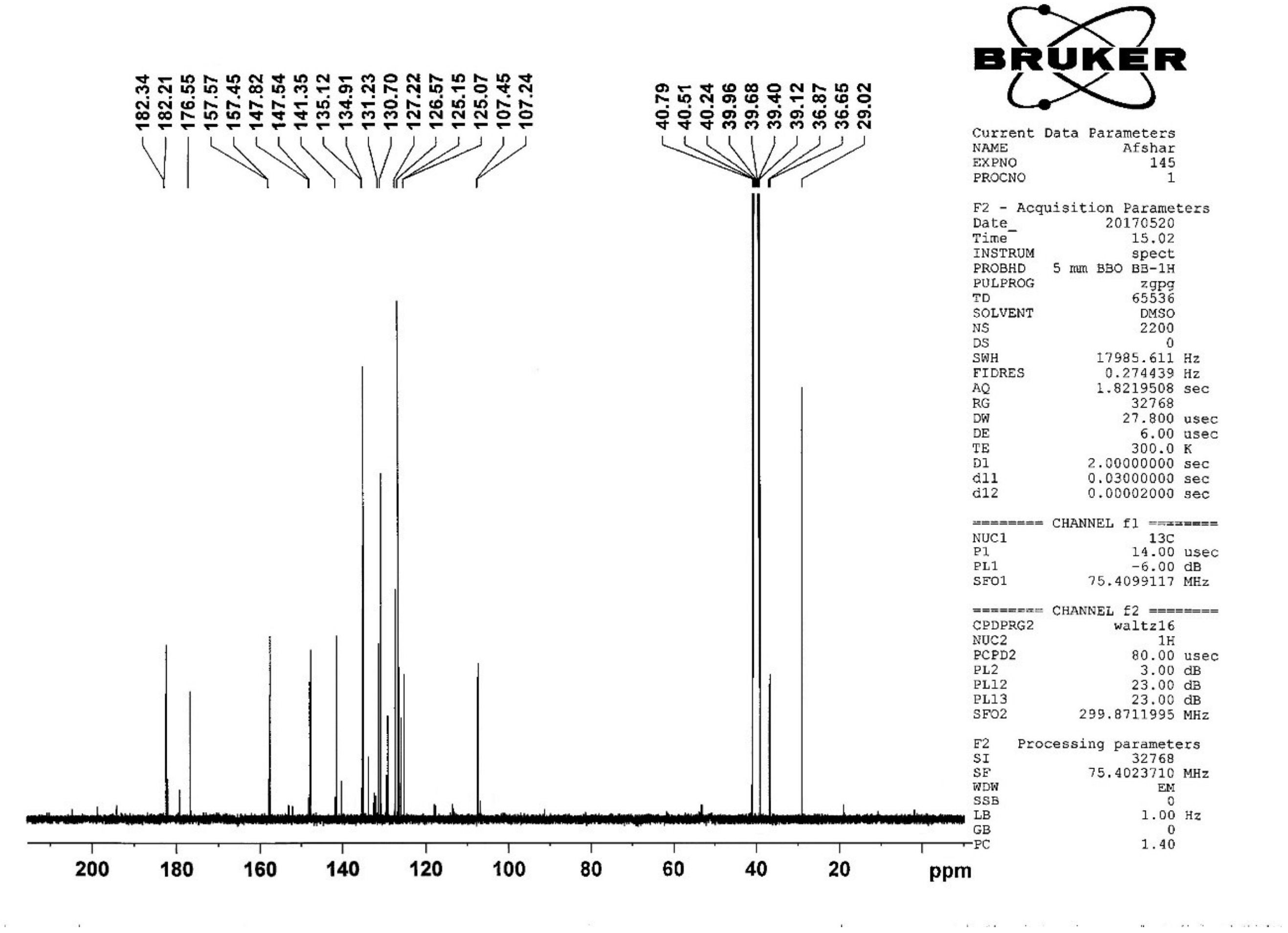

^{13}C NMR of 6a

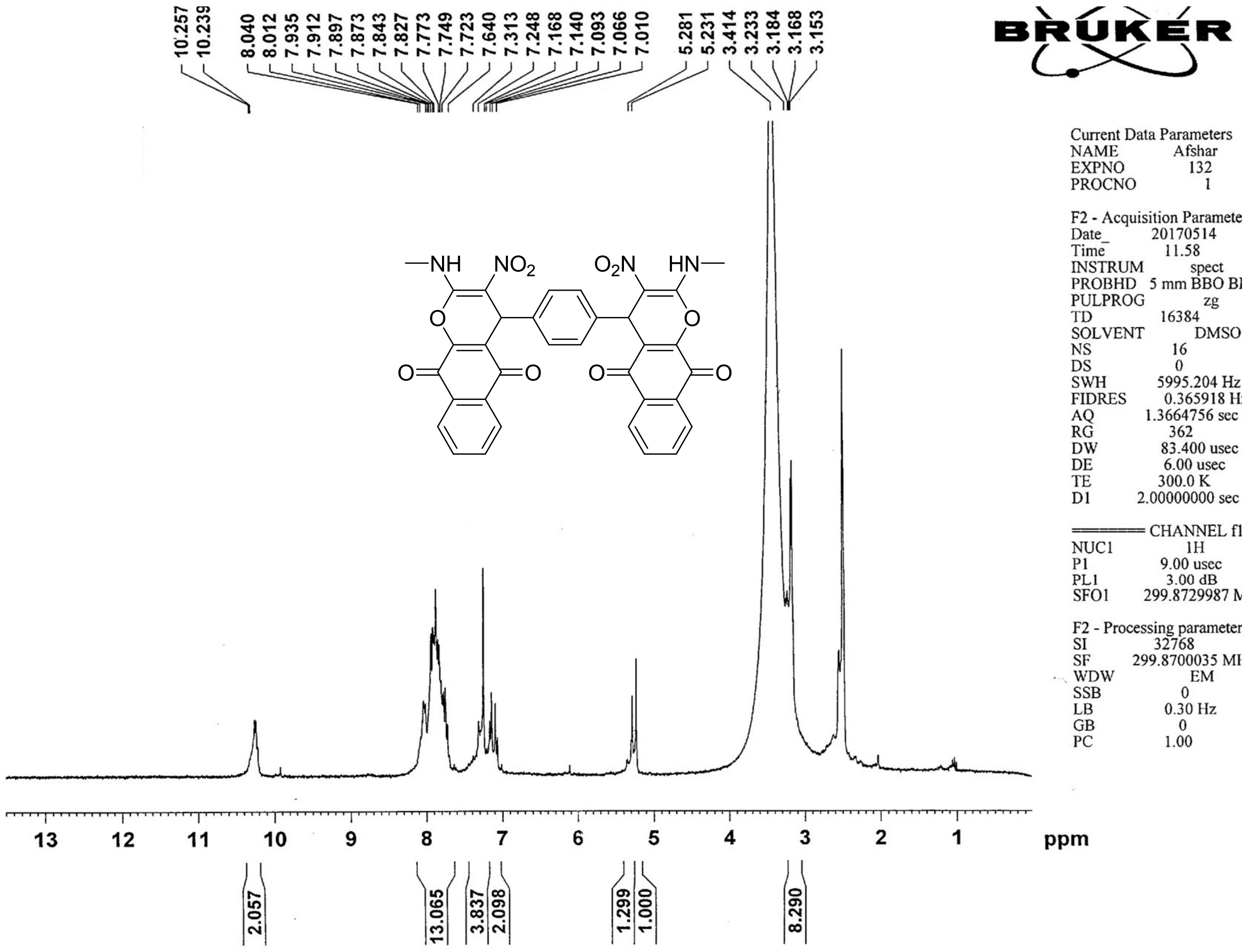
10.257
10.239
8.040
8.012
7.935
7.912
7.897
7.873
7.843
7.827
7.773
7.749
7.723
7.640
7.313
7.248
7.168
7.140
7.093
7.066
7.010
5.281
5.231
3.414
3.233
3.184
3.168
3.153
BRUKER
Current Data Parameters
NAME Afshar
EXPNO 132
PROCNO 1
F2 - Acquisition Paramete
Date_ 20170514
Time 11.58
INSTRUM spect
PROBHD 5 mm BBO BI
PULPROG zg
TD 16384
SOLVENT DMSO
NS 16
DS 0
SWH 5995.204 Hz
FIDRES 0.365918 H
AQ 1.3664756 sec
RG 362
DW 83.400 usec
DE 6.00 usec
TE 300.0 K
D1 2.00000000 sec
CHANNEL f1
NUC1 1H
P1 9.00 usec
PL1 3.00 dB
SFO1 299.8729987 M
F2 - Processing parameter
SI 32768
SF 299.8700035 MI
WDW EM
SSB 0
LB 0.30 Hz
GB 0
PC 1.00
NH
NO2
O2N
HN
O
13 12 11 10 9 8 7 6 5 4 3 2 1 ppm
2.057
13.065
3.837
2.098
1.299
1.000
8.290

^{1}H NMR of 6b

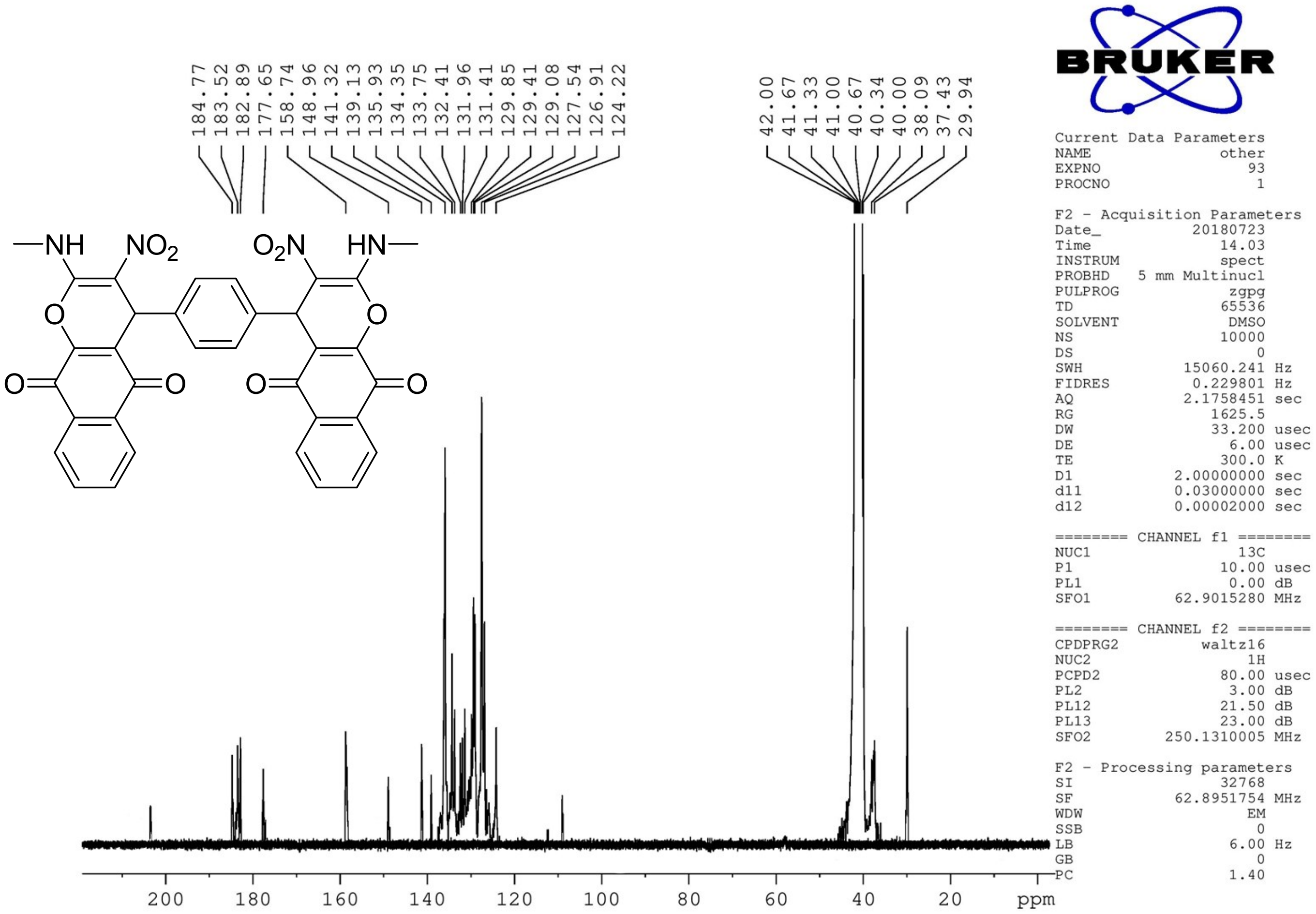
184.77
183.52
182.89
177.65
158.74
148.96
141.32
139.13
135.93
134.35
133.75
132.41
131.96
131.41
129.85
129.41
129.08
127.54
126.91
124.22
42.00
41.67
41.33
41.00
40.67
40.34
40.00
38.09
37.43
29.94
—NH
NO2
O2N
HN—
O
O
O
O
O
O
BRUKER
Current Data Parameters
NAME other
EXPNO 93
PROCNO 1
F2 - Acquisition Parameters
Date_ 20180723
Time 14.03
INSTRUM spect
PROBHD 5 mm Multinucl
PULPROG zgpg
TD 65536
SOLVENT DMSO
NS 10000
DS 0
SWH 15060.241 Hz
FIDRES 0.229801 Hz
AQ 2.1758451 sec
RG 1625.5
DW 33.200 usec
DE 6.00 usec
TE 300.0 K
D1 2.00000000 sec
d11 0.03000000 sec
d12 0.00002000 sec
======== CHANNEL f1 ========
NUC1 13C
P1 10.00 usec
PL1 0.00 dB
SFO1 62.9015280 MHz
======== CHANNEL f2 ========
CPDPRG2 waltz16
NUC2 1H
PCPD2 80.00 usec
PL2 3.00 dB
PL12 21.50 dB
PL13 23.00 dB
SFO2 250.1310005 MHz
F2 - Processing parameters
SI 32768
SF 62.8951754 MHz
WDW EM
SSB 0
LB 6.00 Hz
GB 0
PC 1.40
200
180
160
140
120
100
80
60
40
20
ppm

^{13}C NMR of 6b

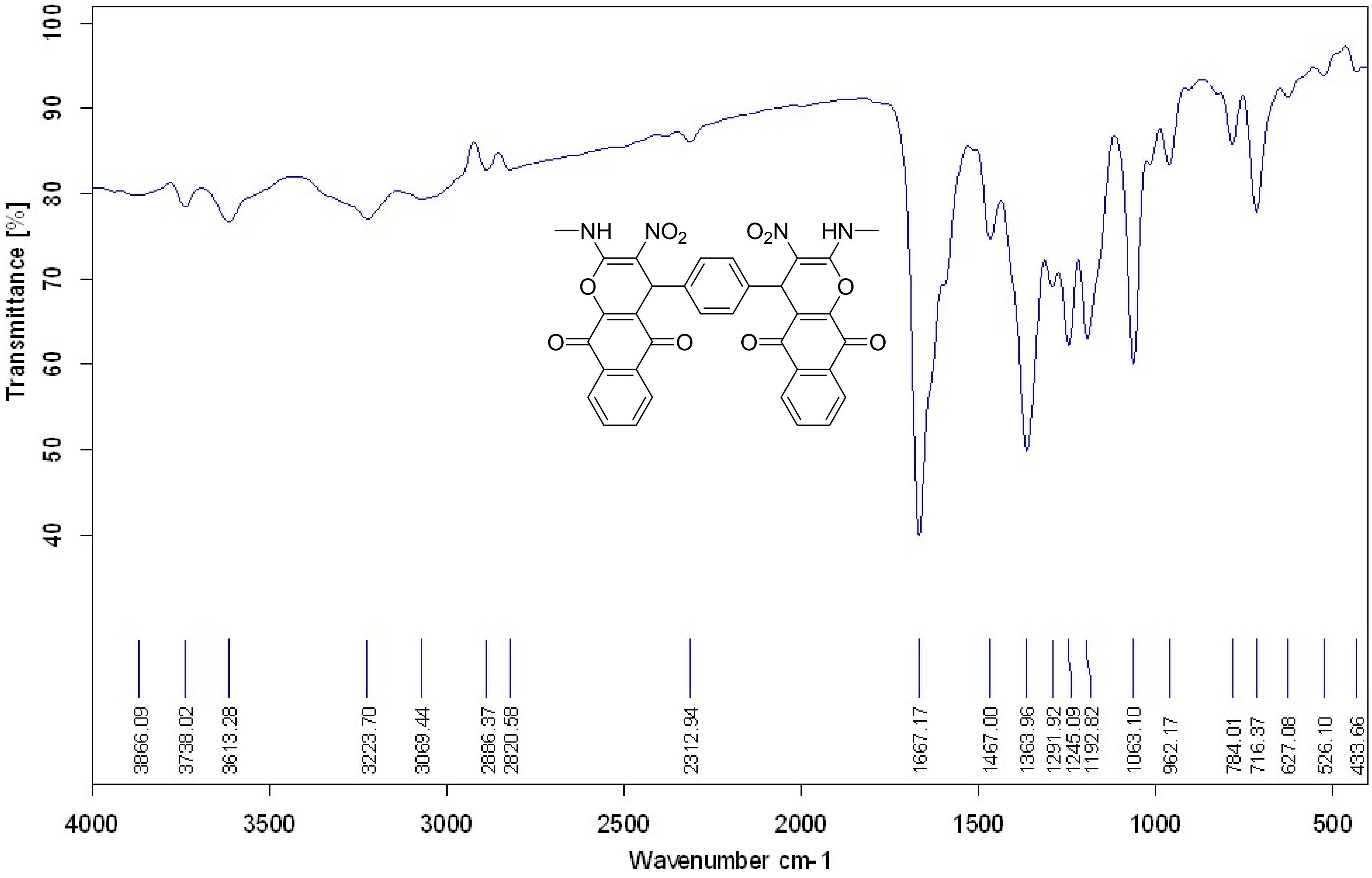
Transmittance [%]
100
90
80
70
60
50
40
—NH
NO2
O2N
HN—
O
O
O
O
O
O
3866.09
3738.02
3613.28
3223.70
3069.44
2886.37
2820.58
2312.94
1667.17
1467.00
1363.96
1291.92
1245.09
1192.82
1063.10
962.17
784.01
716.37
627.08
526.10
433.66
4000
3500
3000
2500
2000
1500
1000
500
Wavenumber cm-1

IR of 6b

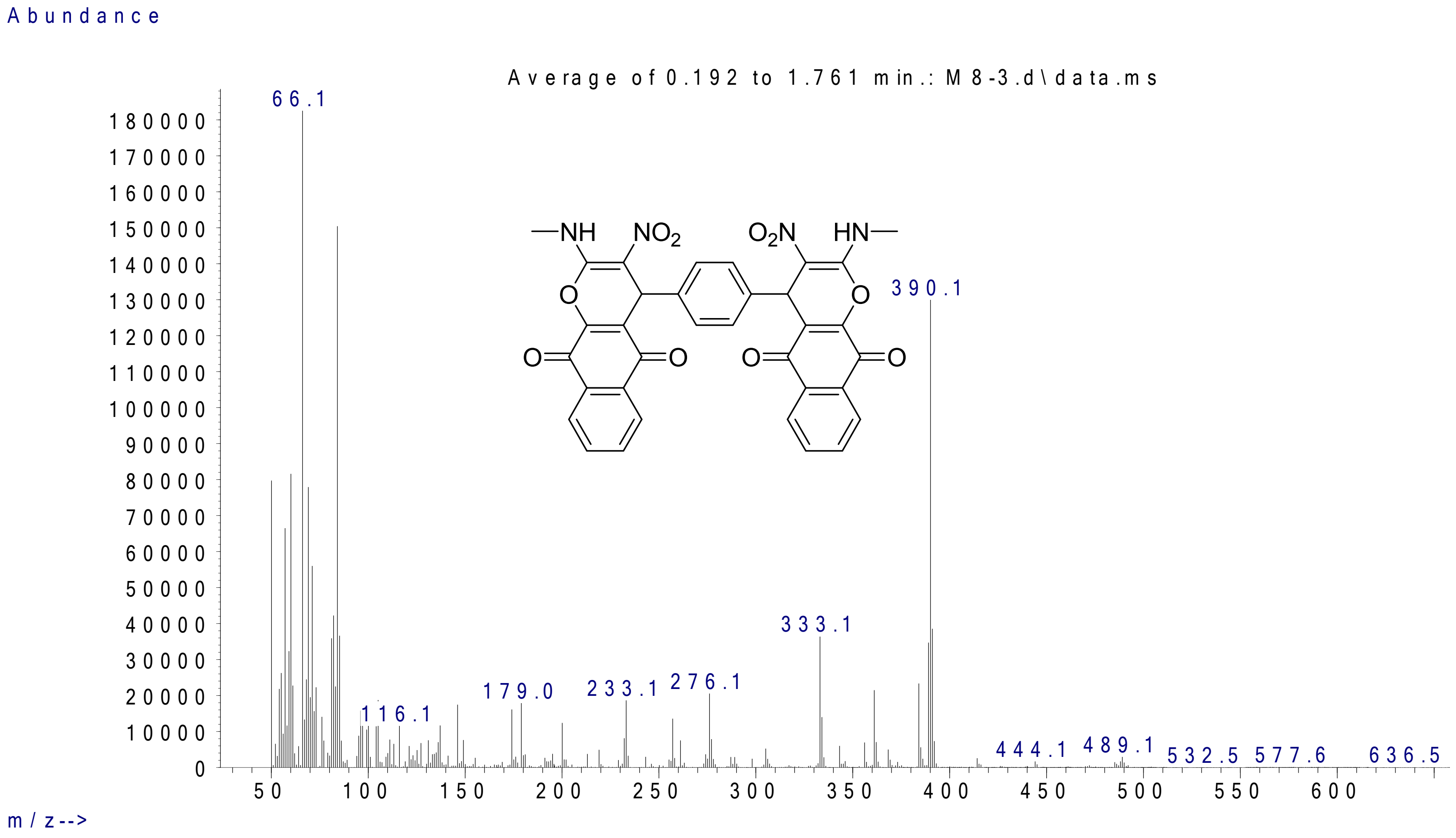
Abundance
Average of 0.192 to 1.761 min.: M8-3.d\data.ms
66.1
390.1
333.1
276.1
233.1
179.0
116.1
444.1
489.1
532.5
577.6
636.5
180000
170000
160000
150000
140000
130000
120000
110000
100000
90000
80000
70000
60000
50000
40000
30000
20000
10000
0
50
100
150
200
250
300
350
400
450
500
550
600
m/z-->
NH
NO2
O2N
HN
O

Mass of 6b

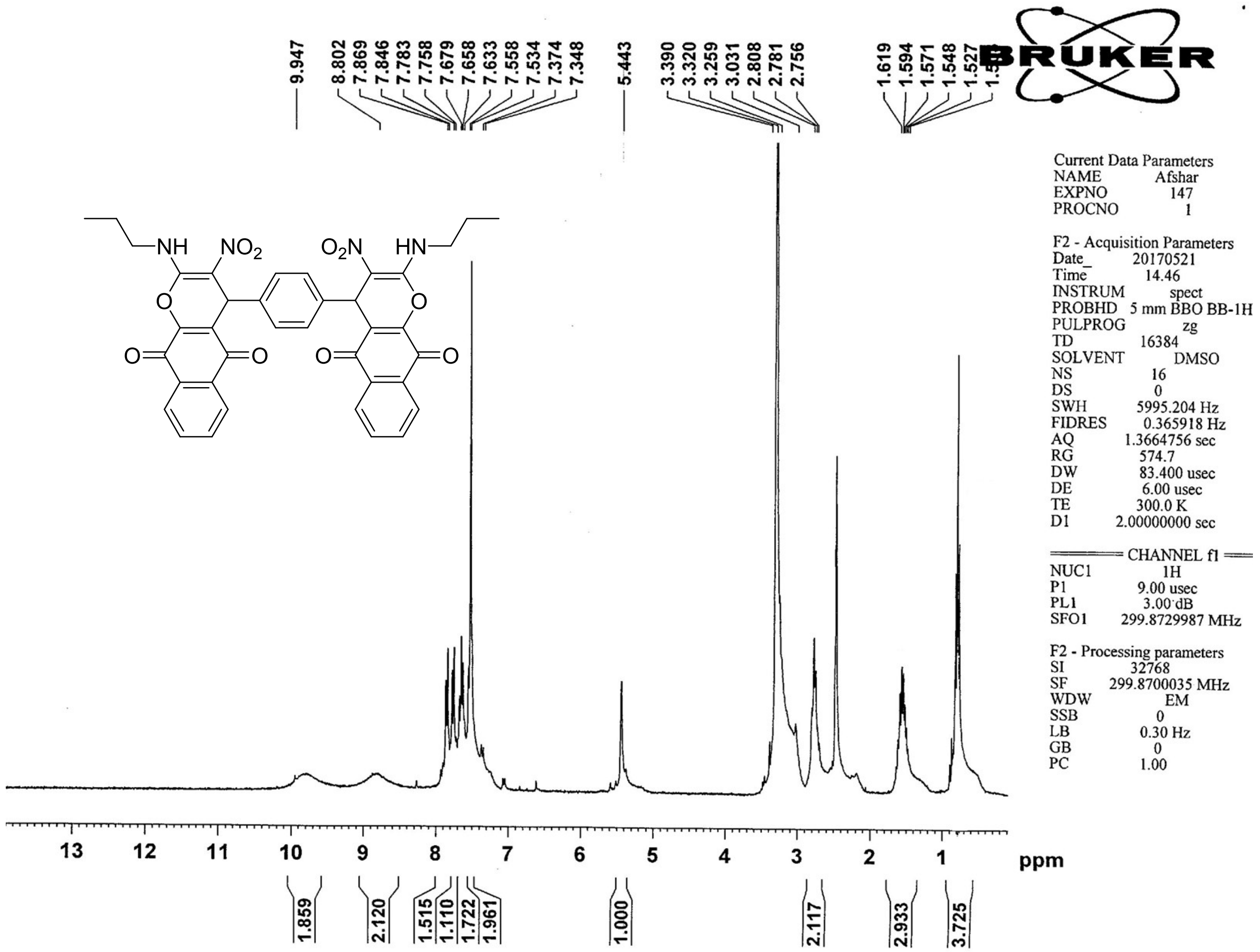
9.947
8.802
7.869
7.846
7.783
7.758
7.679
7.658
7.633
7.558
7.534
7.374
7.348
5.443
3.390
3.320
3.259
3.031
2.808
2.781
2.756
1.619
1.594
1.571
1.548
1.527
BRUKER
NH
NO2
O2N
HN
O
Current Data Parameters
NAME Afshar
EXPNO 147
PROCNO 1
F2 - Acquisition Parameters
Date_ 20170521
Time 14.46
INSTRUM spect
PROBHD 5 mm BBO BB-1H
PULPROG zg
TD 16384
SOLVENT DMSO
NS 16
DS 0
SWH 5995.204 Hz
FIDRES 0.365918 Hz
AQ 1.3664756 sec
RG 574.7
DW 83.400 usec
DE 6.00 usec
TE 300.0 K
D1 2.00000000 sec
CHANNEL f1
NUC1 1H
P1 9.00 usec
PL1 3.00 dB
SFO1 299.8729987 MHz
F2 - Processing parameters
SI 32768
SF 299.8700035 MHz
WDW EM
SSB 0
LB 0.30 Hz
GB 0
PC 1.00
13 12 11 10 9 8 7 6 5 4 3 2 1 ppm
1.859
2.120
1.515
1.110
1.722
1.961
1.000
2.117
2.933
3.725

^{1}H NMR of 6c

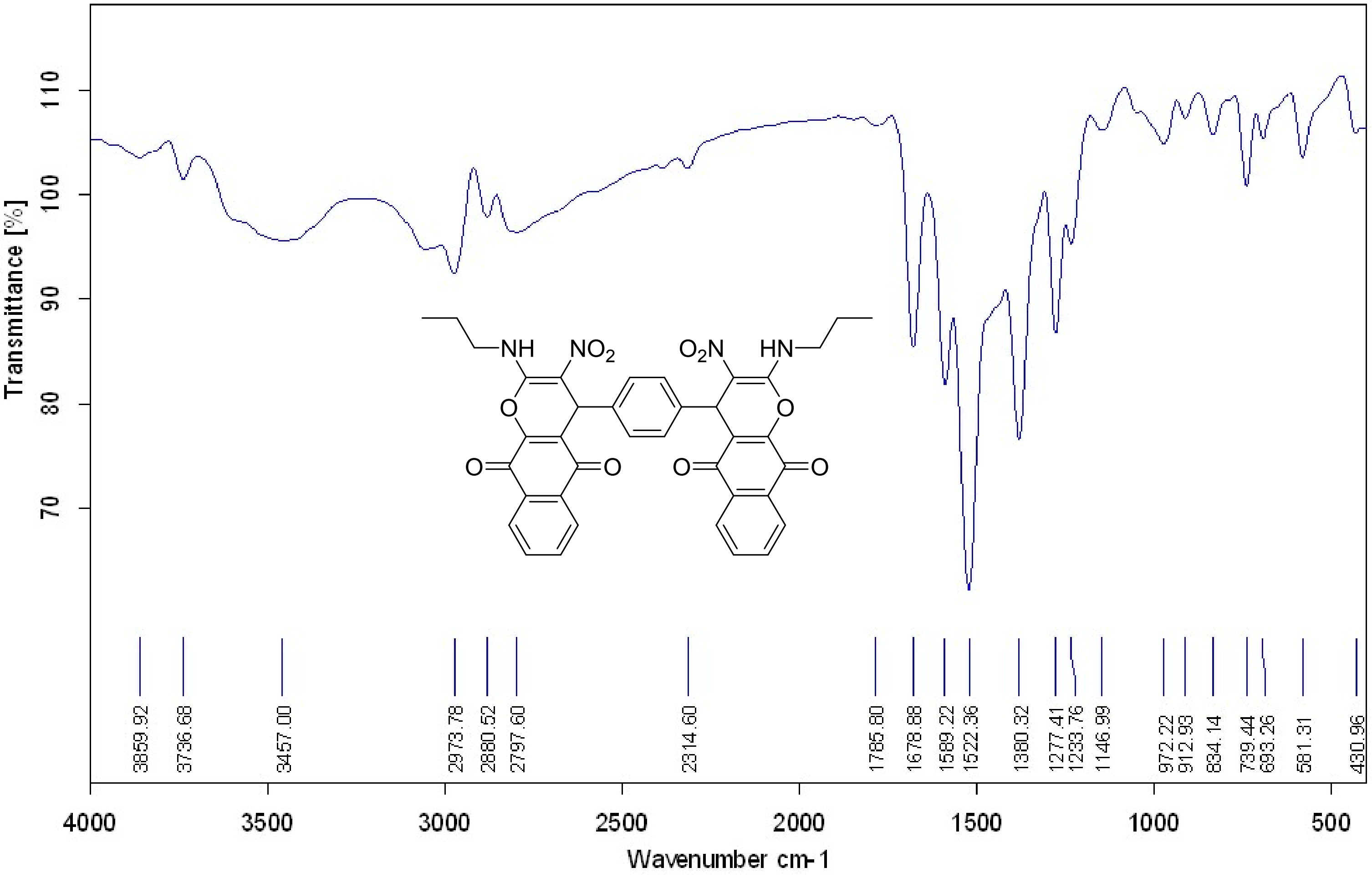
Transmittance [%]
110
100
90
80
70
NH
NO2
O2N
HN
O
O
O
O
O
O
3859.92
3736.68
3457.00
2973.78
2880.52
2797.60
2314.60
1785.80
1678.88
1589.22
1522.36
1380.32
1277.41
1233.76
1146.99
972.22
912.93
834.14
739.44
693.26
581.31
430.96
4000
3500
3000
2500
2000
1500
1000
500
Wavenumber cm-1

IR of 6c

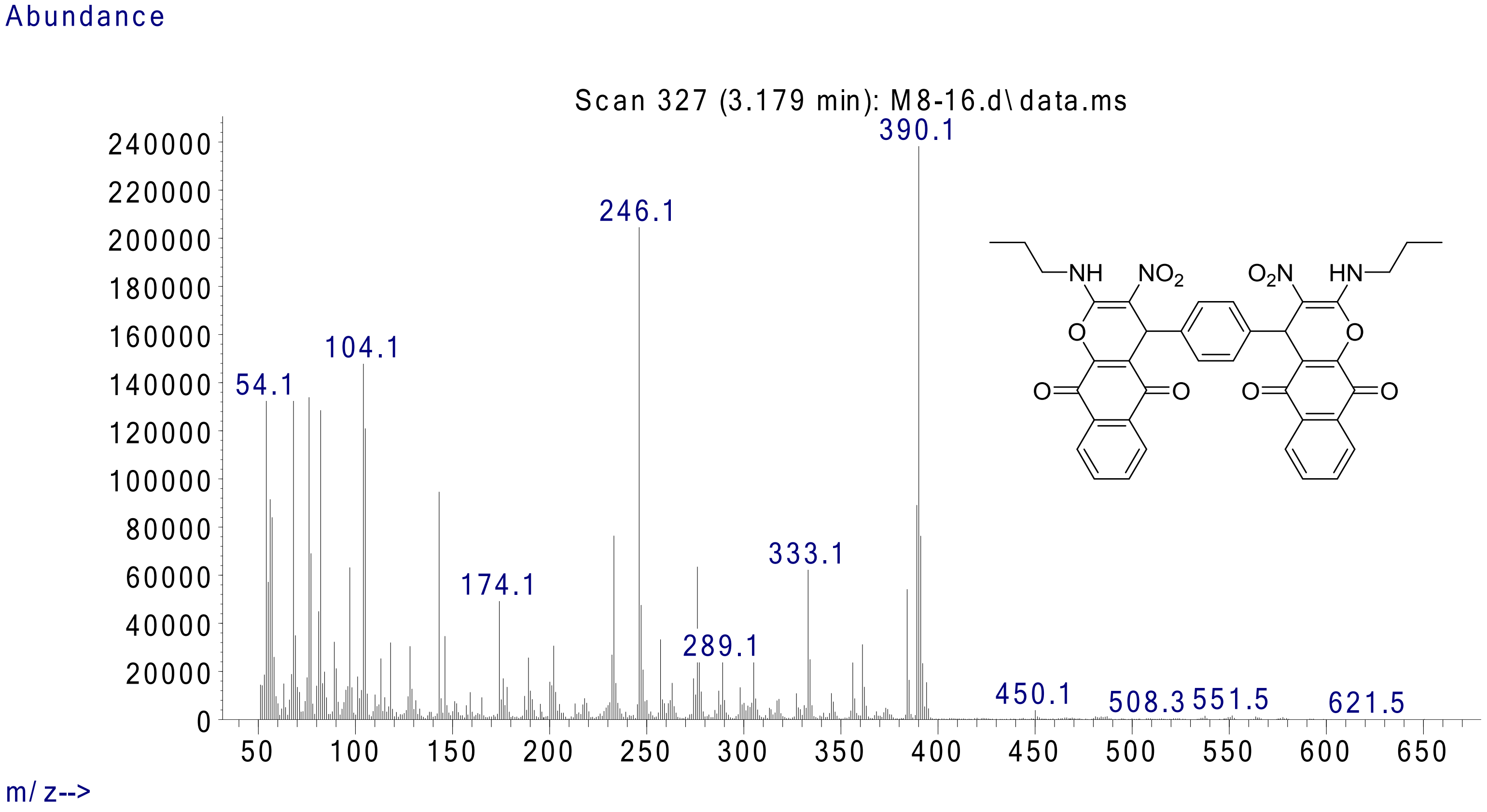
Abundance
Scan 327 (3.179 min): M8-16.d\data.ms
240000
220000
200000
180000
160000
140000
120000
100000
80000
60000
40000
20000
0
390.1
246.1
104.1
54.1
174.1
333.1
289.1
450.1
508.3
551.5
621.5
50
100
150
200
250
300
350
400
450
500
550
600
650
m/z-->
NH
NO2
O2N
HN
O
O
O
O
O
O

Mass of 6c

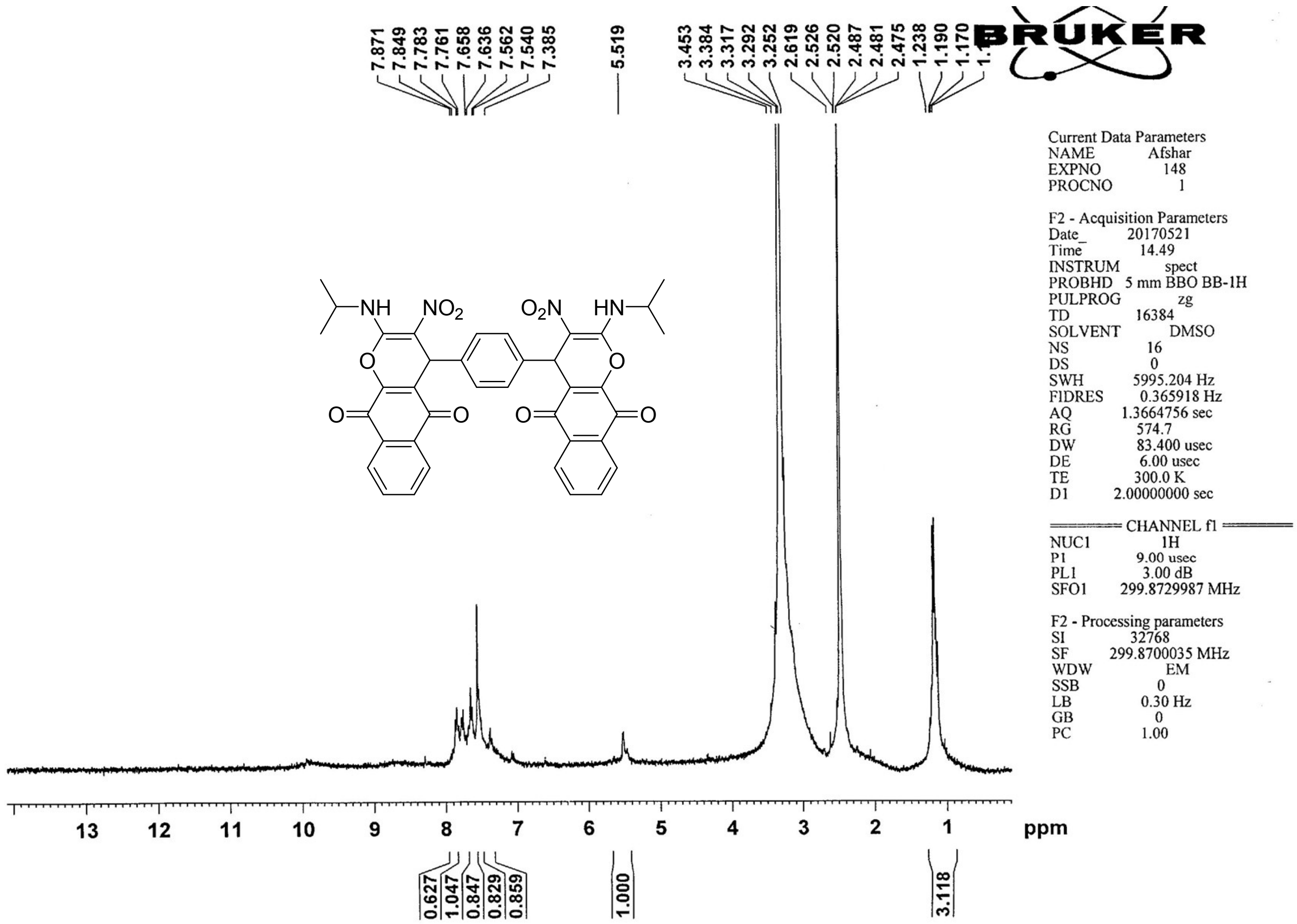
7.871
7.849
7.783
7.761
7.658
7.636
7.562
7.540
7.385
5.519
3.453
3.384
3.317
3.292
3.252
2.619
2.526
2.520
2.487
2.481
2.475
1.238
1.190
1.170
BRUKER
Current Data Parameters
NAME Afshar
EXPNO 148
PROCNO 1
F2 - Acquisition Parameters
Date_ 20170521
Time 14.49
INSTRUM spect
PROBHD 5 mm BBO BB-1H
PULPROG zg
TD 16384
SOLVENT DMSO
NS 16
DS 0
SWH 5995.204 Hz
FIDRES 0.365918 Hz
AQ 1.3664756 sec
RG 574.7
DW 83.400 usec
DE 6.00 usec
TE 300.0 K
D1 2.00000000 sec
CHANNEL f1
NUC1 1H
P1 9.00 usec
PL1 3.00 dB
SFO1 299.8729987 MHz
F2 - Processing parameters
SI 32768
SF 299.8700035 MHz
WDW EM
SSB 0
LB 0.30 Hz
GB 0
PC 1.00
NH
NO2
O2N
HN
O
13 12 11 10 9 8 7 6 5 4 3 2 1 ppm
0.627
1.047
0.847
0.829
0.859
1.000
3.118

^{1}H NMR of 6d

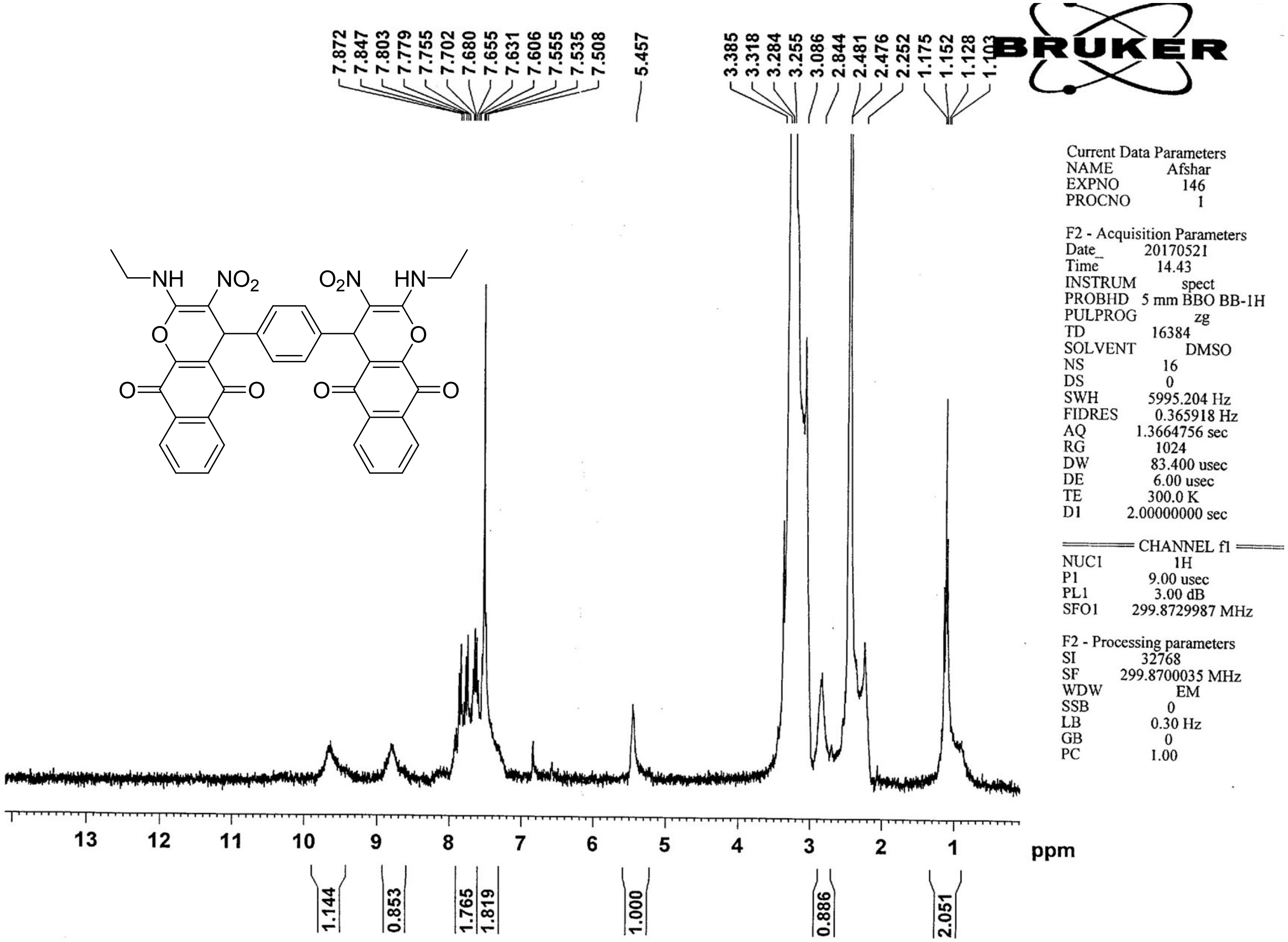
7.872
7.847
7.803
7.779
7.755
7.702
7.680
7.655
7.631
7.606
7.555
7.535
7.508
5.457
3.385
3.318
3.284
3.255
3.086
2.844
2.481
2.476
2.252
1.175
1.152
1.128
BRUKER
NH
NO2
O2N
HN
O
Current Data Parameters
NAME Afshar
EXPNO 146
PROCNO 1
F2 - Acquisition Parameters
Date_ 20170521
Time 14.43
INSTRUM spect
PROBHD 5 mm BBO BB-1H
PULPROG zg
TD 16384
SOLVENT DMSO
NS 16
DS 0
SWH 5995.204 Hz
FIDRES 0.365918 Hz
AQ 1.3664756 sec
RG 1024
DW 83.400 usec
DE 6.00 usec
TE 300.0 K
D1 2.00000000 sec
CHANNEL f1
NUC1 1H
P1 9.00 usec
PL1 3.00 dB
SFO1 299.8729987 MHz
F2 - Processing parameters
SI 32768
SF 299.8700035 MHz
WDW EM
SSB 0
LB 0.30 Hz
GB 0
PC 1.00
13
12
11
10
9
8
7
6
5
4
3
2
1
ppm
1.144
0.853
1.765
1.819
1.000
0.886
2.051

^{1}H NMR of 6e

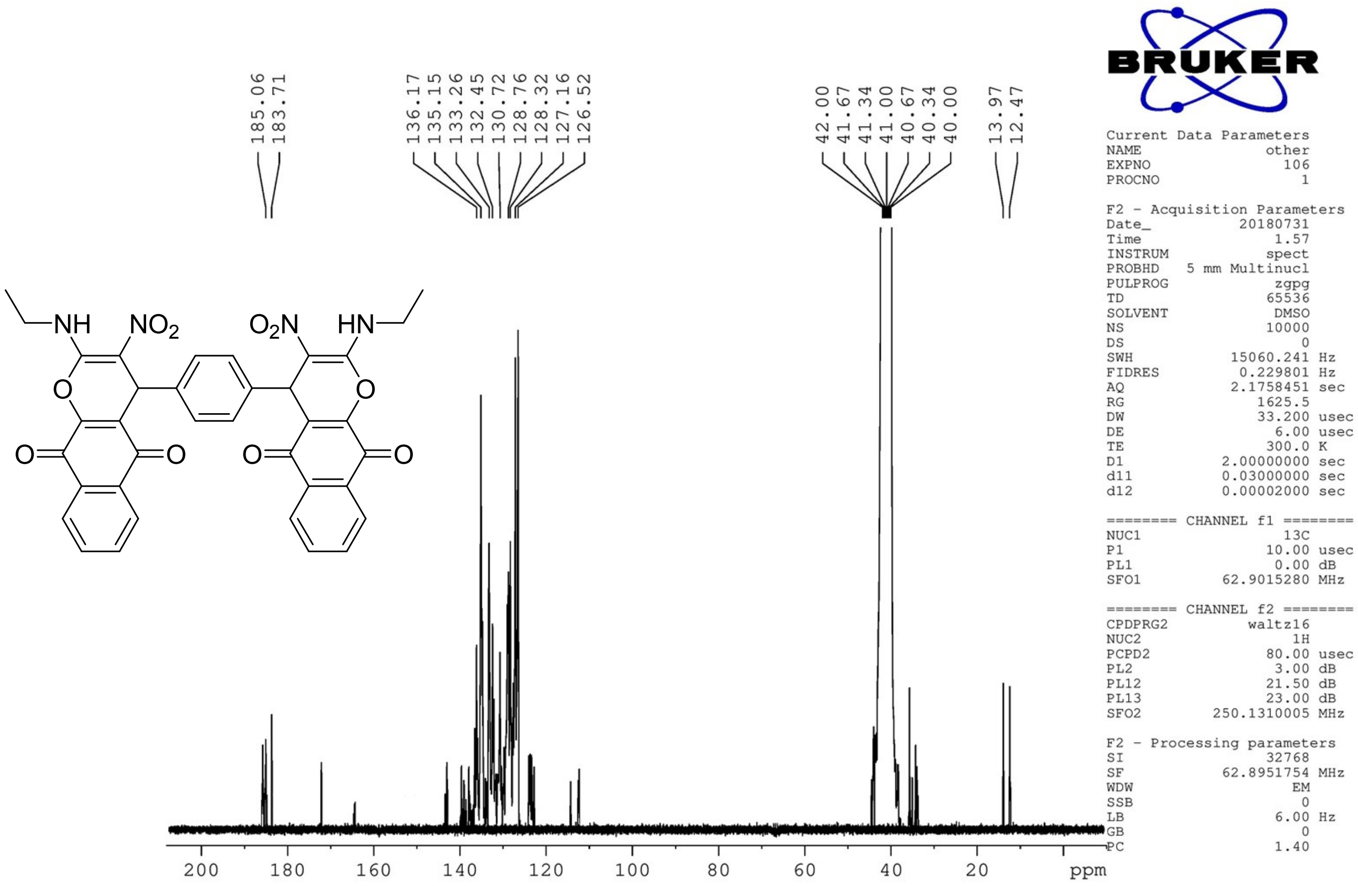
NH
NO2
O2N
HN
O
O
O
O
O
O
185.06
183.71
136.17
135.15
133.26
132.45
130.72
128.76
128.32
127.16
126.52
42.00
41.67
41.34
41.00
40.67
40.34
40.00
13.97
12.47
BRUKER
Current Data Parameters
NAME other
EXPNO 106
PROCNO 1
F2 - Acquisition Parameters
Date_ 20180731
Time 1.57
INSTRUM spect
PROBHD 5 mm Multinucl
PULPROG zgpg
TD 65536
SOLVENT DMSO
NS 10000
DS 0
SWH 15060.241 Hz
FIDRES 0.229801 Hz
AQ 2.1758451 sec
RG 1625.5
DW 33.200 usec
DE 6.00 usec
TE 300.0 K
D1 2.00000000 sec
d11 0.03000000 sec
d12 0.00002000 sec
======== CHANNEL f1 ========
NUC1 13C
P1 10.00 usec
PL1 0.00 dB
SFO1 62.9015280 MHz
======== CHANNEL f2 ========
CPDPRG2 waltz16
NUC2 1H
PCPD2 80.00 usec
PL2 3.00 dB
PL12 21.50 dB
PL13 23.00 dB
SFO2 250.1310005 MHz
F2 - Processing parameters
SI 32768
SF 62.8951754 MHz
WDW EM
SSB 0
LB 6.00 Hz
GB 0
PC 1.40
200
180
160
140
120
100
80
60
40
20
ppm

^{13}C NMR of 6e

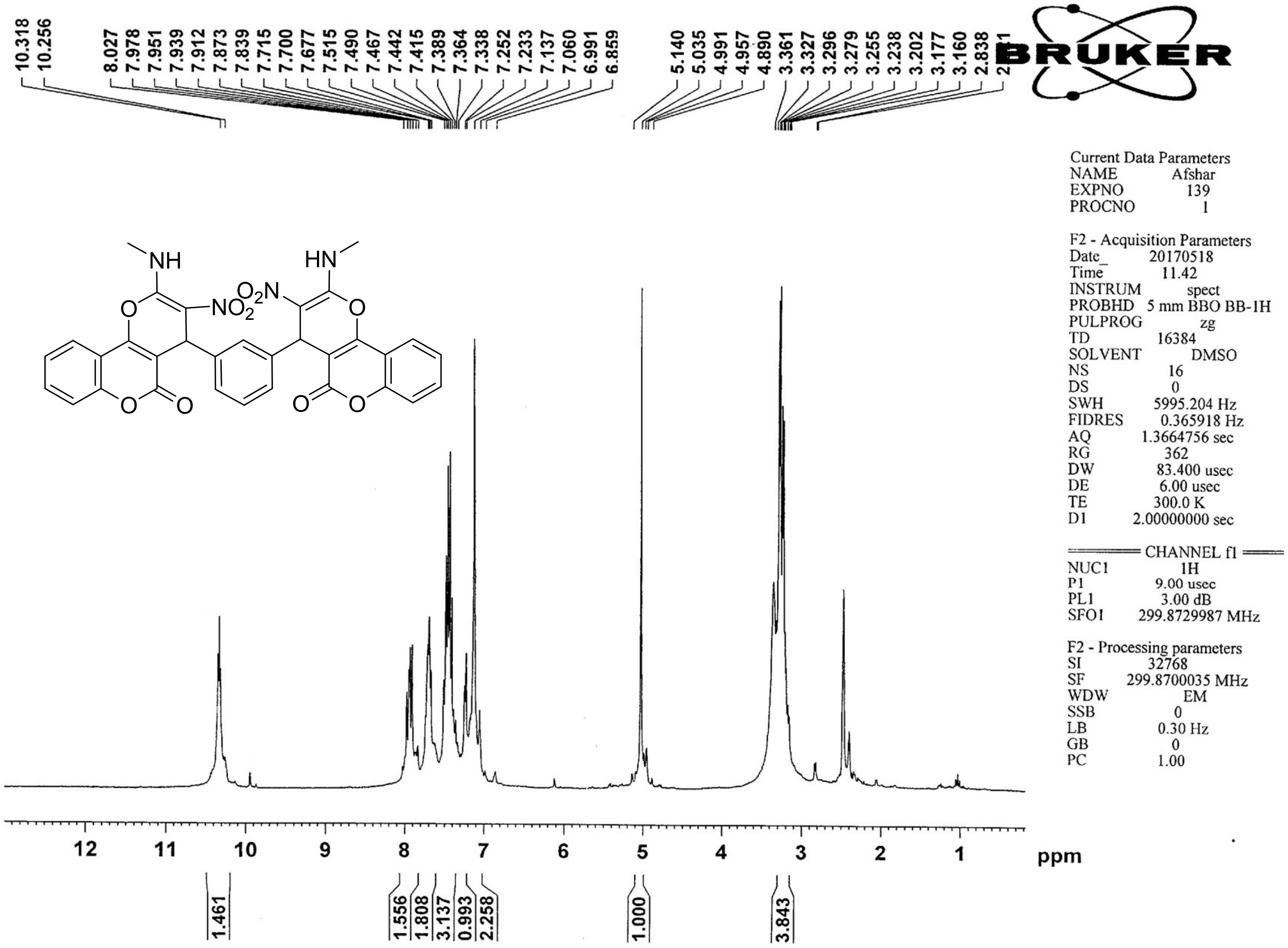

^{1}H NMR of 7a

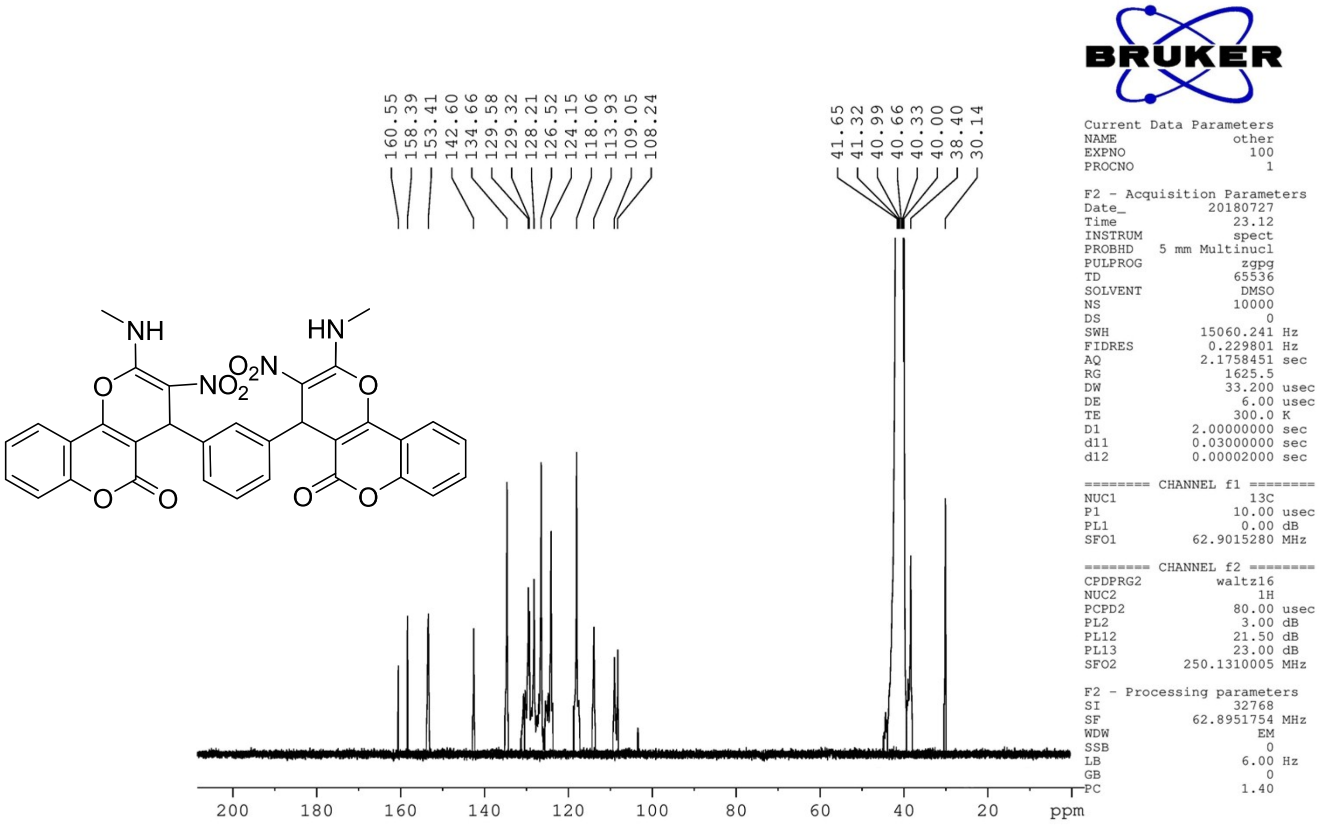
160.55
158.39
153.41
142.60
134.66
129.58
129.32
128.21
126.52
124.15
118.06
113.93
109.05
108.24
41.65
41.32
40.99
40.66
40.33
40.00
38.40
30.14
NH
HN
O_2N
NO_2
O
BRUKER
Current Data Parameters
NAME other
EXPNO 100
PROCNO 1
F2 - Acquisition Parameters
Date_ 20180727
Time 23.12
INSTRUM spect
PROBHD 5 mm Multinucl
PULPROG zgpg
TD 65536
SOLVENT DMSO
NS 10000
DS 0
SWH 15060.241 Hz
FIDRES 0.229801 Hz
AQ 2.1758451 sec
RG 1625.5
DW 33.200 usec
DE 6.00 usec
TE 300.0 K
D1 2.00000000 sec
d11 0.03000000 sec
d12 0.00002000 sec
======== CHANNEL f1 ========
NUC1 13C
P1 10.00 usec
PL1 0.00 dB
SFO1 62.9015280 MHz
======== CHANNEL f2 ========
CPDPRG2 waltz16
NUC2 1H
PCPD2 80.00 usec
PL2 3.00 dB
PL12 21.50 dB
PL13 23.00 dB
SFO2 250.1310005 MHz
F2 - Processing parameters
SI 32768
SF 62.8951754 MHz
WDW EM
SSB 0
LB 6.00 Hz
GB 0
PC 1.40
200 180 160 140 120 100 80 60 40 20 ppm

^{13}C NMR of 7a

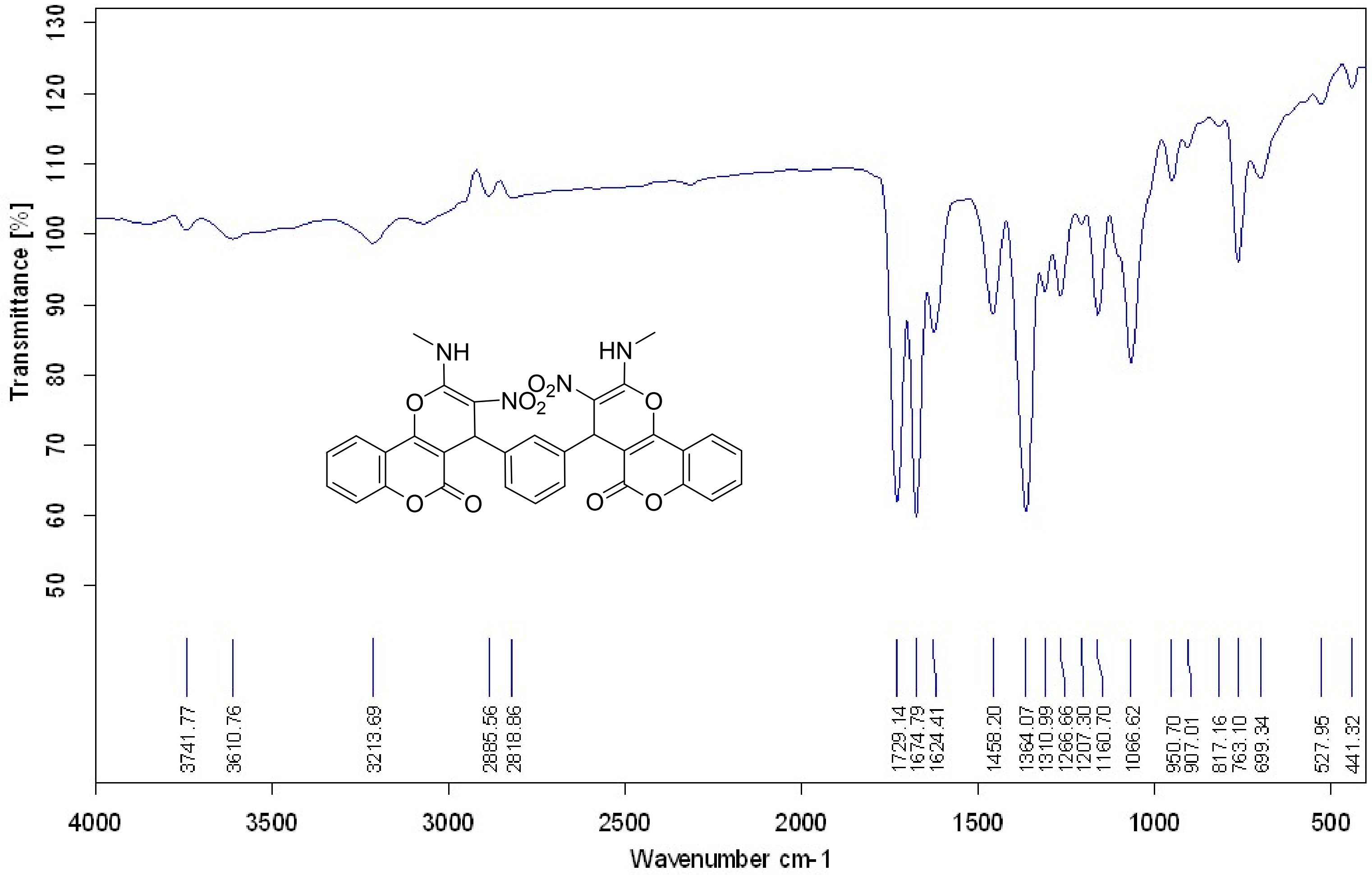
Transmittance [%]
Wavenumber cm-1
3741.77
3610.76
3213.69
2885.56
2818.86
1729.14
1674.79
1624.41
1458.20
1364.07
1310.99
1266.66
1207.30
1160.70
1066.62
950.70
907.01
817.16
763.10
699.34
527.95
441.32

IR of 7a

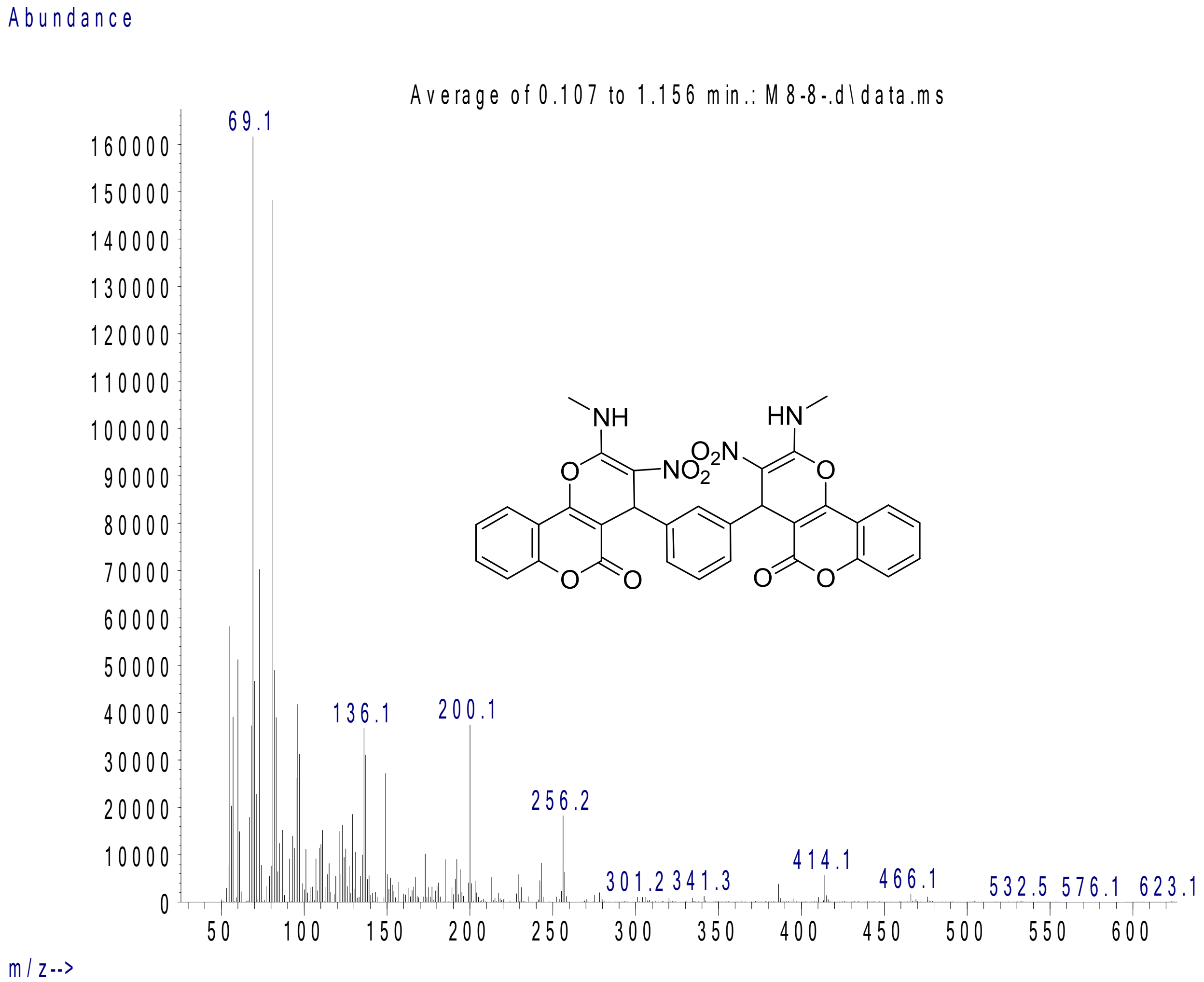
Abundance
Average of 0.107 to 1.156 min.: M8-8-.d\data.ms
69.1
136.1
200.1
256.2
301.2
341.3
414.1
466.1
532.5
576.1
623.1
160000
150000
140000
130000
120000
110000
100000
90000
80000
70000
60000
50000
40000
30000
20000
10000
0
50
100
150
200
250
300
350
400
450
500
550
600
m/z-->
NH
HN
NO_2
O_2N
O
O
O
O
O
O

Mass of 7a

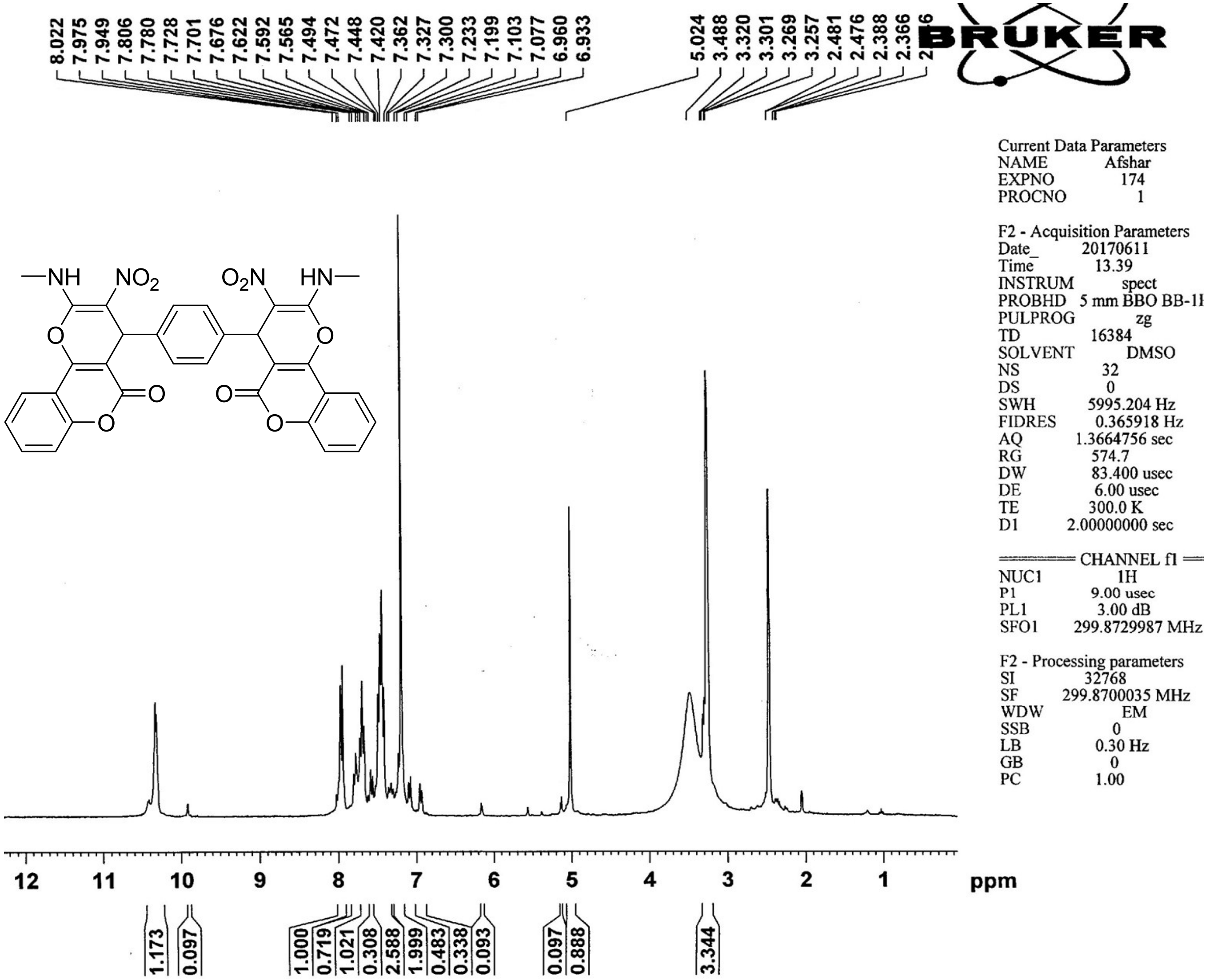
8.022 7.975 7.949 7.806 7.780 7.728 7.701 7.676 7.622 7.592 7.565 7.494 7.472 7.448 7.420 7.362 7.327 7.300 7.233 7.199 7.103 7.077 6.960 6.933
5.024 3.488 3.320 3.301 3.269 3.257 2.481 2.476 2.388 2.366
BRUKER
Current Data Parameters
NAME Afshar
EXPNO 174
PROCNO 1
F2 - Acquisition Parameters
Date_ 20170611
Time 13.39
INSTRUM spect
PROBHD 5 mm BBO BB-1I
PULPROG zg
TD 16384
SOLVENT DMSO
NS 32
DS 0
SWH 5995.204 Hz
FIDRES 0.365918 Hz
AQ 1.3664756 sec
RG 574.7
DW 83.400 usec
DE 6.00 usec
TE 300.0 K
D1 2.00000000 sec
CHANNEL f1
NUC1 1H
P1 9.00 usec
PL1 3.00 dB
SFO1 299.8729987 MHz
F2 - Processing parameters
SI 32768
SF 299.8700035 MHz
WDW EM
SSB 0
LB 0.30 Hz
GB 0
PC 1.00
NH
NO_2
O_2N
HN
O
O
O
O
O
O
12 11 10 9 8 7 6 5 4 3 2 1 ppm
1.173 0.097 1.000 0.719 1.021 0.308 2.588 1.999 0.483 0.338 0.093 0.097 0.888 3.344

^{1}H NMR of 7b

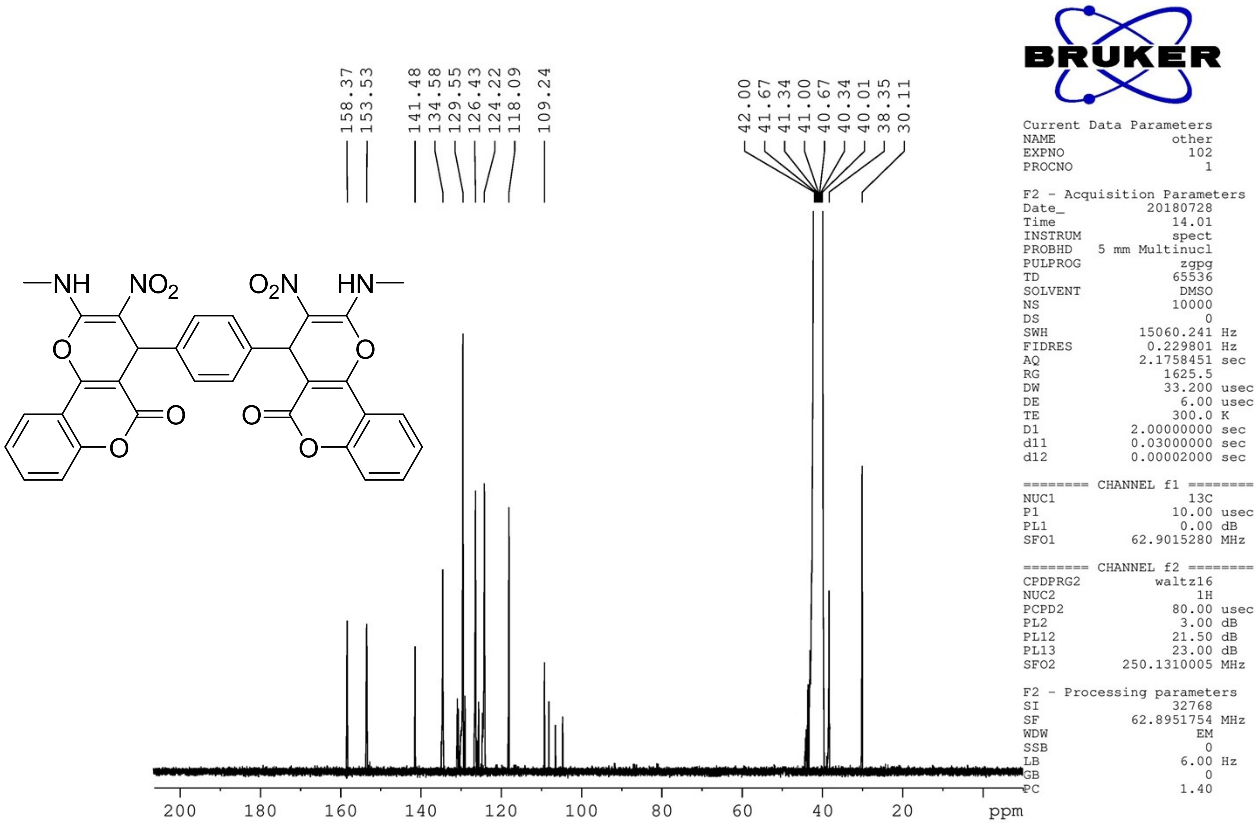
158.37
153.53
141.48
134.58
129.55
126.43
124.22
118.09
109.24
42.00
41.67
41.34
41.00
40.67
40.34
40.01
38.35
30.11
BRUKER
Current Data Parameters
NAME other
EXPNO 102
PROCNO 1
F2 - Acquisition Parameters
Date_ 20180728
Time 14.01
INSTRUM spect
PROBHD 5 mm Multinucl
PULPROG zgpg
TD 65536
SOLVENT DMSO
NS 10000
DS 0
SWH 15060.241 Hz
FIDRES 0.229801 Hz
AQ 2.1758451 sec
RG 1625.5
DW 33.200 usec
DE 6.00 usec
TE 300.0 K
D1 2.00000000 sec
d11 0.03000000 sec
d12 0.00002000 sec
======== CHANNEL f1 ========
NUC1 13C
P1 10.00 usec
PL1 0.00 dB
SFO1 62.9015280 MHz
======== CHANNEL f2 ========
CPDPRG2 waltz16
NUC2 1H
PCPD2 80.00 usec
PL2 3.00 dB
PL12 21.50 dB
PL13 23.00 dB
SFO2 250.1310005 MHz
F2 - Processing parameters
SI 32768
SF 62.8951754 MHz
WDW EM
SSB 0
LB 6.00 Hz
GB 0
PC 1.40
200 180 160 140 120 100 80 60 40 20 ppm

^{13}C NMR of 7b

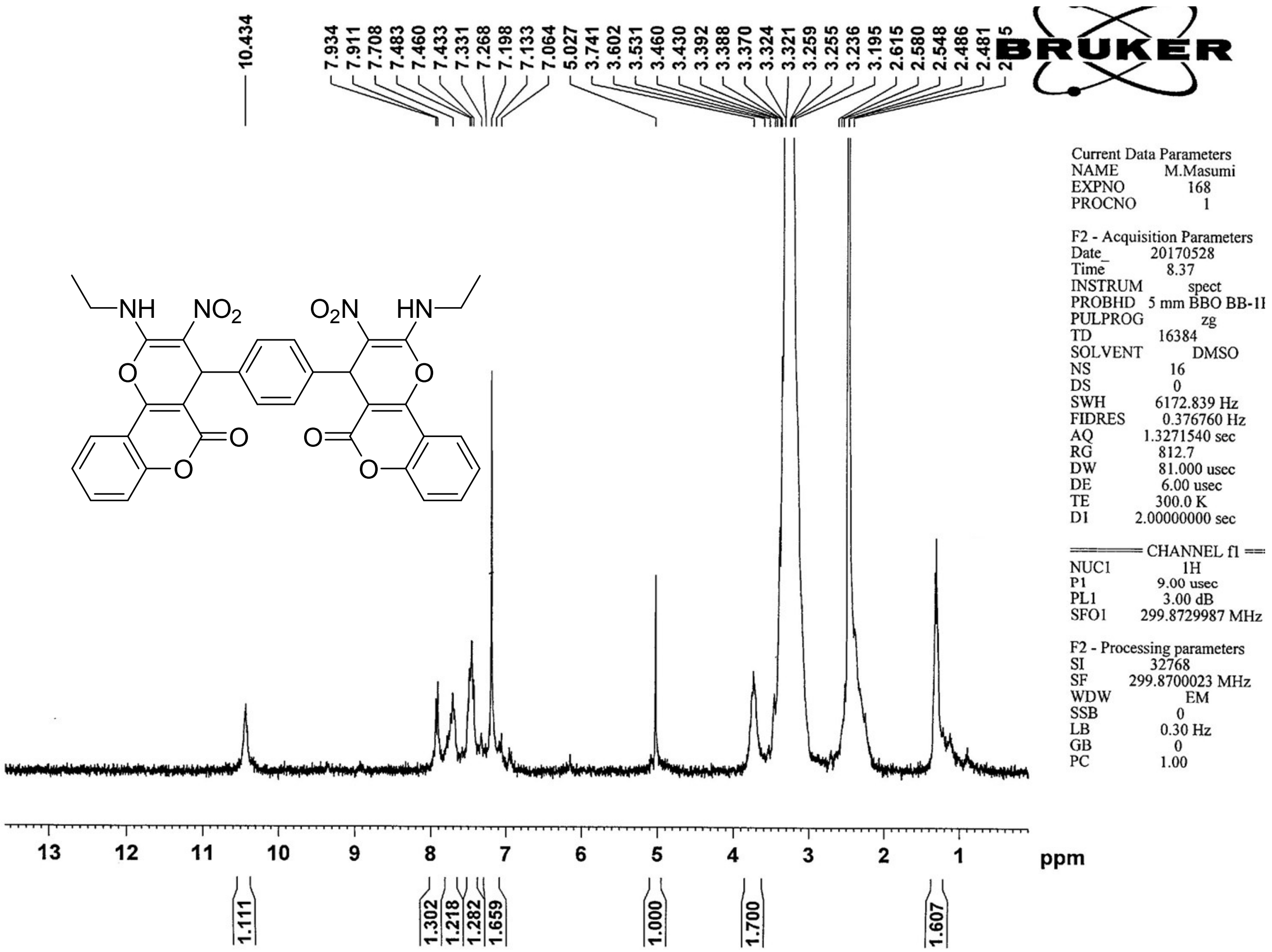

10.434
7.934
7.911
7.708
7.483
7.460
7.433
7.331
7.268
7.198
7.133
7.064
5.027
3.741
3.602
3.531
3.460
3.430
3.392
3.388
3.370
3.324
3.321
3.259
3.255
3.236
3.195
2.615
2.580
2.548
2.486
2.481
BRUKER
NH
NO2
O2N
HN
O
O
O
O
O
O
Current Data Parameters
NAME M.Masumi
EXPNO 168
PROCNO 1
F2 - Acquisition Parameters
Date_ 20170528
Time 8.37
INSTRUM spect
PROBHD 5 mm BBO BB-1H
PULPROG zg
TD 16384
SOLVENT DMSO
NS 16
DS 0
SWH 6172.839 Hz
FIDRES 0.376760 Hz
AQ 1.3271540 sec
RG 812.7
DW 81.000 usec
DE 6.00 usec
TE 300.0 K
D1 2.00000000 sec
CHANNEL f1
NUC1 1H
P1 9.00 usec
PL1 3.00 dB
SFO1 299.8729987 MHz
F2 - Processing parameters
SI 32768
SF 299.8700023 MHz
WDW EM
SSB 0
LB 0.30 Hz
GB 0
PC 1.00
13 12 11 10 9 8 7 6 5 4 3 2 1 ppm
1.111
1.302
1.218
1.282
1.659
1.000
1.700
1.607

^{1}H NMR of 7c

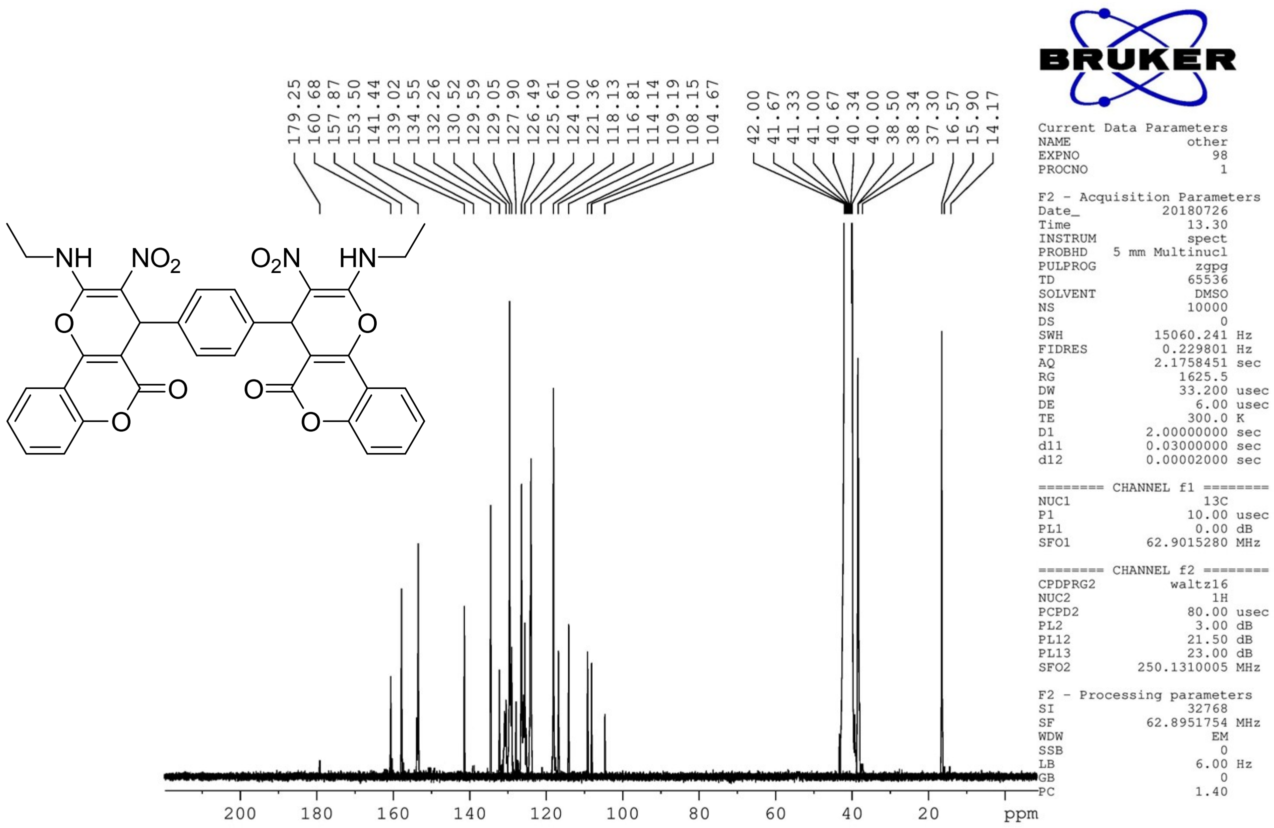

NH
NO2
O2N
HN
O
O
O
O
O
O
179.25
160.68
157.87
153.50
141.44
139.02
134.55
132.26
130.52
129.59
129.05
127.90
126.49
125.61
124.00
121.36
118.13
116.81
114.14
109.19
108.15
104.67
42.00
41.67
41.33
41.00
40.67
40.34
40.00
38.50
38.34
37.30
16.57
15.90
14.17
200
180
160
140
120
100
80
60
40
20
ppm
BRUKER
Current Data Parameters
NAME other
EXPNO 98
PROCNO 1
F2 - Acquisition Parameters
Date_ 20180726
Time 13.30
INSTRUM spect
PROBHD 5 mm Multinucl
PULPROG zgpg
TD 65536
SOLVENT DMSO
NS 10000
DS 0
SWH 15060.241 Hz
FIDRES 0.229801 Hz
AQ 2.1758451 sec
RG 1625.5
DW 33.200 usec
DE 6.00 usec
TE 300.0 K
D1 2.00000000 sec
d11 0.03000000 sec
d12 0.00002000 sec
======== CHANNEL f1 ========
NUC1 13C
P1 10.00 usec
PL1 0.00 dB
SFO1 62.9015280 MHz
======== CHANNEL f2 ========
CPDPRG2 waltz16
NUC2 1H
PCPD2 80.00 usec
PL2 3.00 dB
PL12 21.50 dB
PL13 23.00 dB
SFO2 250.1310005 MHz
F2 - Processing parameters
SI 32768
SF 62.8951754 MHz
WDW EM
SSB 0
LB 6.00 Hz
GB 0
PC 1.40

^{13}C NMR of 7c

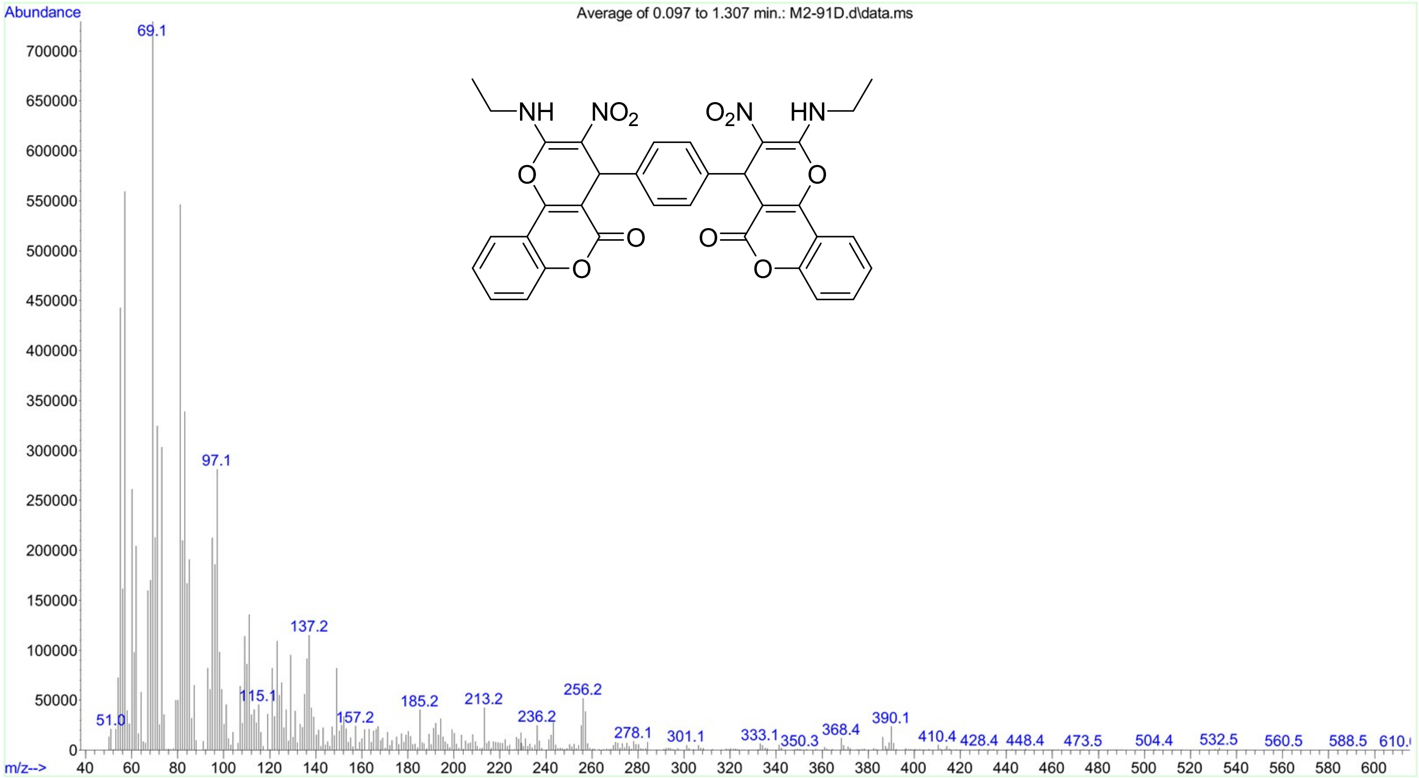
Abundance
Average of 0.097 to 1.307 min.: M2-91D.d\data.ms
69.1
97.1
137.2
115.1
51.0
157.2
185.2
213.2
236.2
256.2
278.1
301.1
333.1
350.3
368.4
390.1
410.4
428.4
448.4
473.5
504.4
532.5
560.5
588.5
m/z-->
NH
NO2
O2N
HN
O
O
O
O
O
O

Mass of 7c

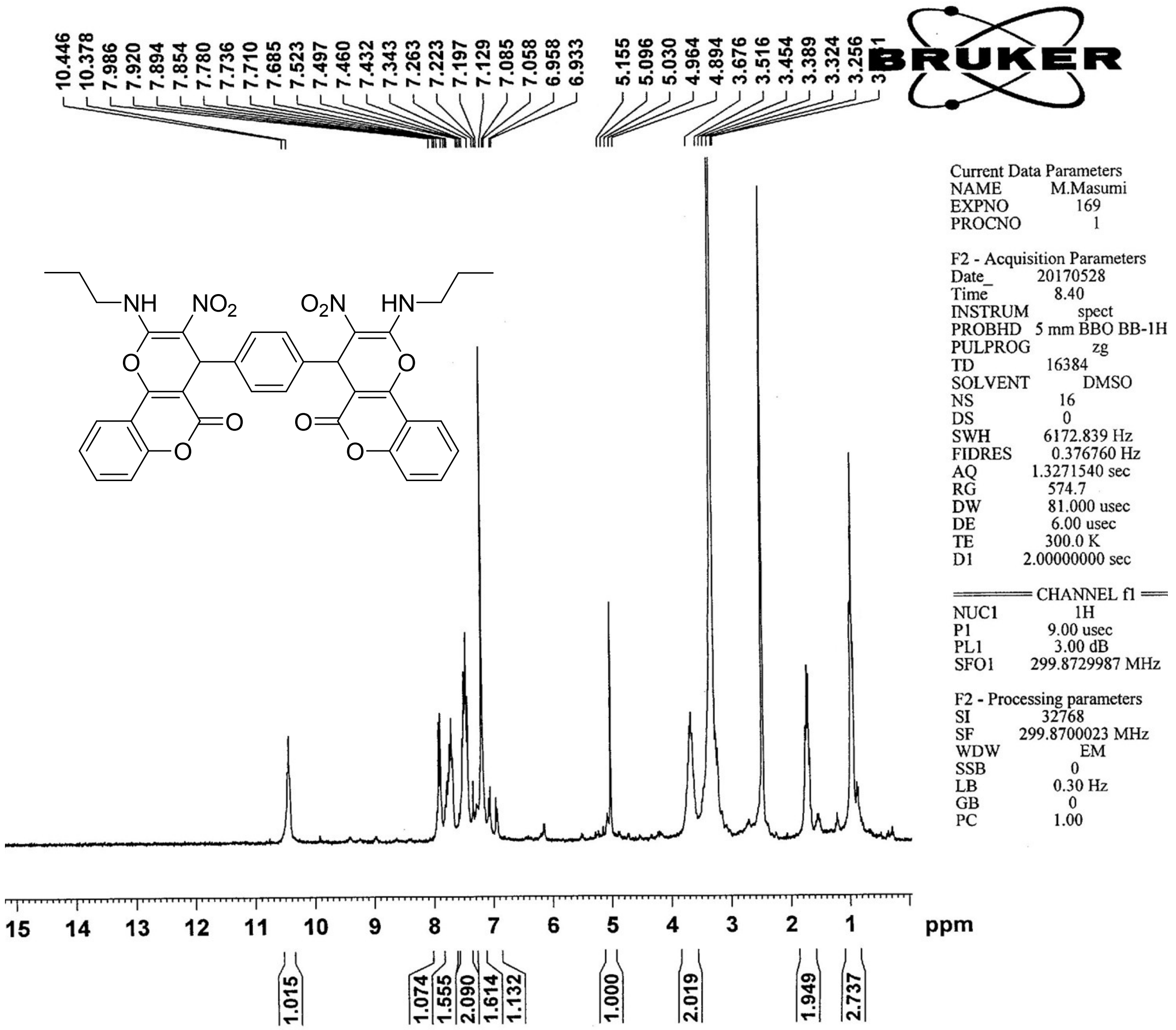
10.446
10.378
7.986
7.920
7.894
7.854
7.780
7.736
7.710
7.685
7.523
7.497
7.460
7.432
7.343
7.263
7.223
7.197
7.129
7.085
7.058
6.958
6.933
5.155
5.096
5.030
4.964
4.894
3.676
3.516
3.454
3.389
3.324
3.256
BRUKER
NH
NO2
O2N
HN
O
O
O
O
O
O
Current Data Parameters
NAME M.Masumi
EXPNO 169
PROCNO 1
F2 - Acquisition Parameters
Date_ 20170528
Time 8.40
INSTRUM spect
PROBHD 5 mm BBO BB-1H
PULPROG zg
TD 16384
SOLVENT DMSO
NS 16
DS 0
SWH 6172.839 Hz
FIDRES 0.376760 Hz
AQ 1.3271540 sec
RG 574.7
DW 81.000 usec
DE 6.00 usec
TE 300.0 K
D1 2.00000000 sec
CHANNEL f1
NUC1 1H
P1 9.00 usec
PL1 3.00 dB
SFO1 299.8729987 MHz
F2 - Processing parameters
SI 32768
SF 299.8700023 MHz
WDW EM
SSB 0
LB 0.30 Hz
GB 0
PC 1.00
15 14 13 12 11 10 9 8 7 6 5 4 3 2 1 ppm
1.015
1.074
1.555
2.090
1.614
1.132
1.000
2.019
1.949
2.737

^{1}H NMR of 7d

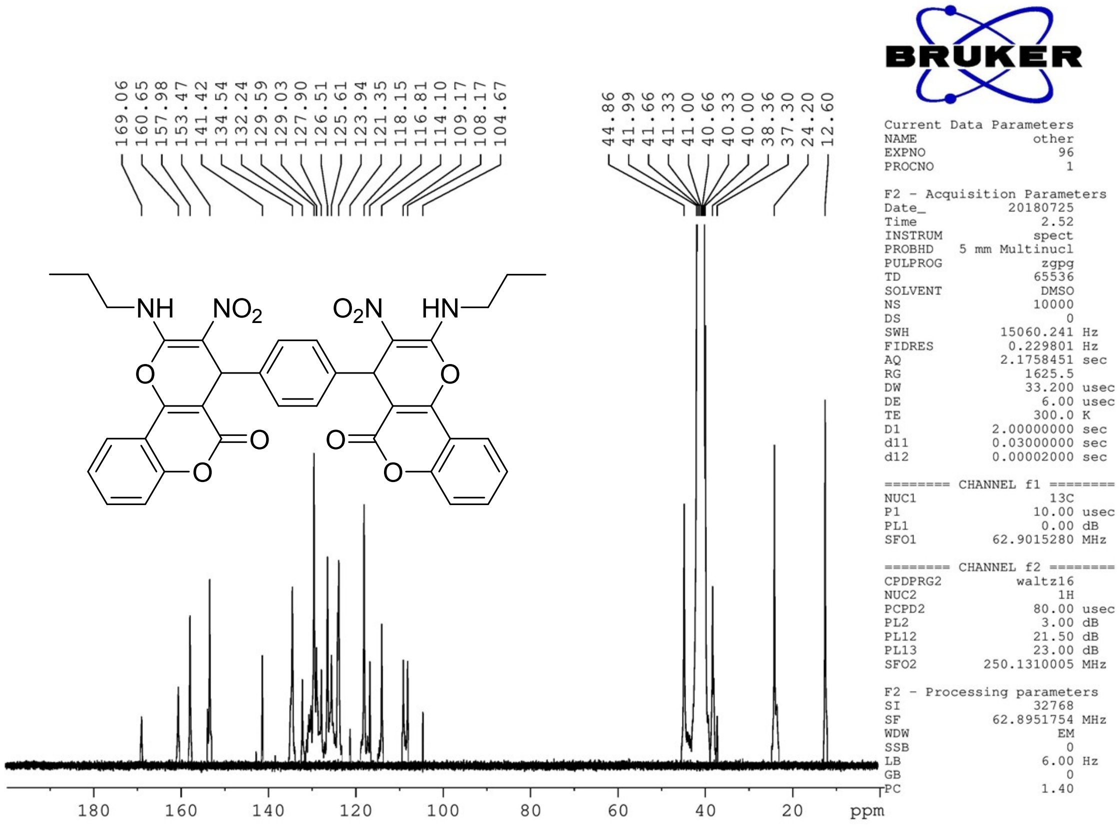
169.06
160.65
157.98
153.47
141.42
134.54
132.24
129.59
129.03
127.90
126.51
125.61
123.94
121.35
118.15
116.81
114.10
109.17
108.17
104.67
44.86
41.99
41.66
41.33
41.00
40.66
40.33
40.00
38.36
37.30
24.20
12.60
NH
NO_2
O_2N
HN
O
BRUKER
Current Data Parameters
NAME other
EXPNO 96
PROCNO 1
F2 - Acquisition Parameters
Date_ 20180725
Time 2.52
INSTRUM spect
PROBHD 5 mm Multinucl
PULPROG zgpg
TD 65536
SOLVENT DMSO
NS 10000
DS 0
SWH 15060.241 Hz
FIDRES 0.229801 Hz
AQ 2.1758451 sec
RG 1625.5
DW 33.200 usec
DE 6.00 usec
TE 300.0 K
D1 2.00000000 sec
d11 0.03000000 sec
d12 0.00002000 sec
======== CHANNEL f1 ========
NUC1 13C
P1 10.00 usec
PL1 0.00 dB
SFO1 62.9015280 MHz
======== CHANNEL f2 ========
CPDPRG2 waltz16
NUC2 1H
PCPD2 80.00 usec
PL2 3.00 dB
PL12 21.50 dB
PL13 23.00 dB
SFO2 250.1310005 MHz
F2 - Processing parameters
SI 32768
SF 62.8951754 MHz
WDW EM
SSB 0
LB 6.00 Hz
GB 0
PC 1.40
180
160
140
120
100
80
60
40
20
ppm

^{13}C NMR of 7d

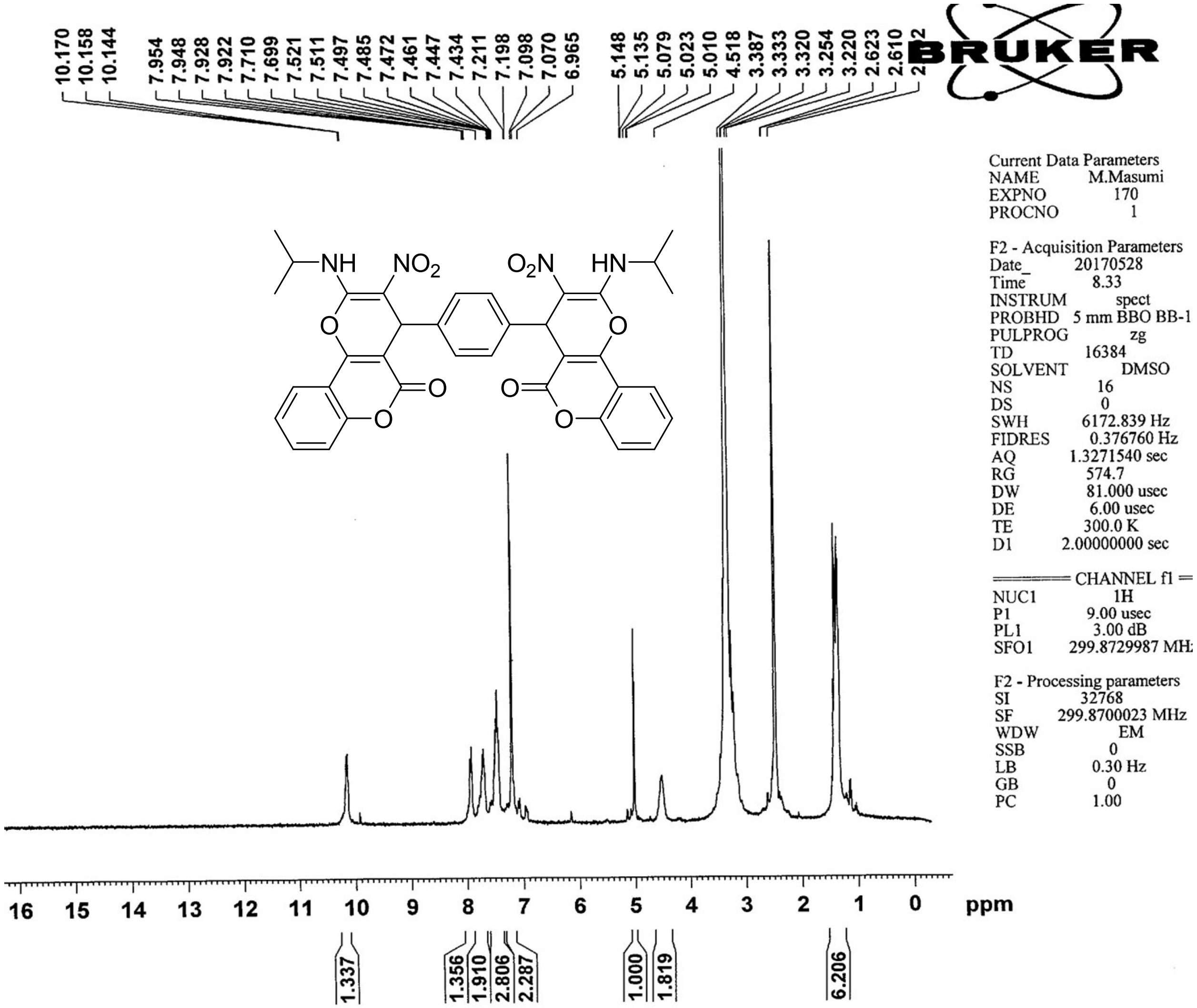

^{1}H NMR of 7e

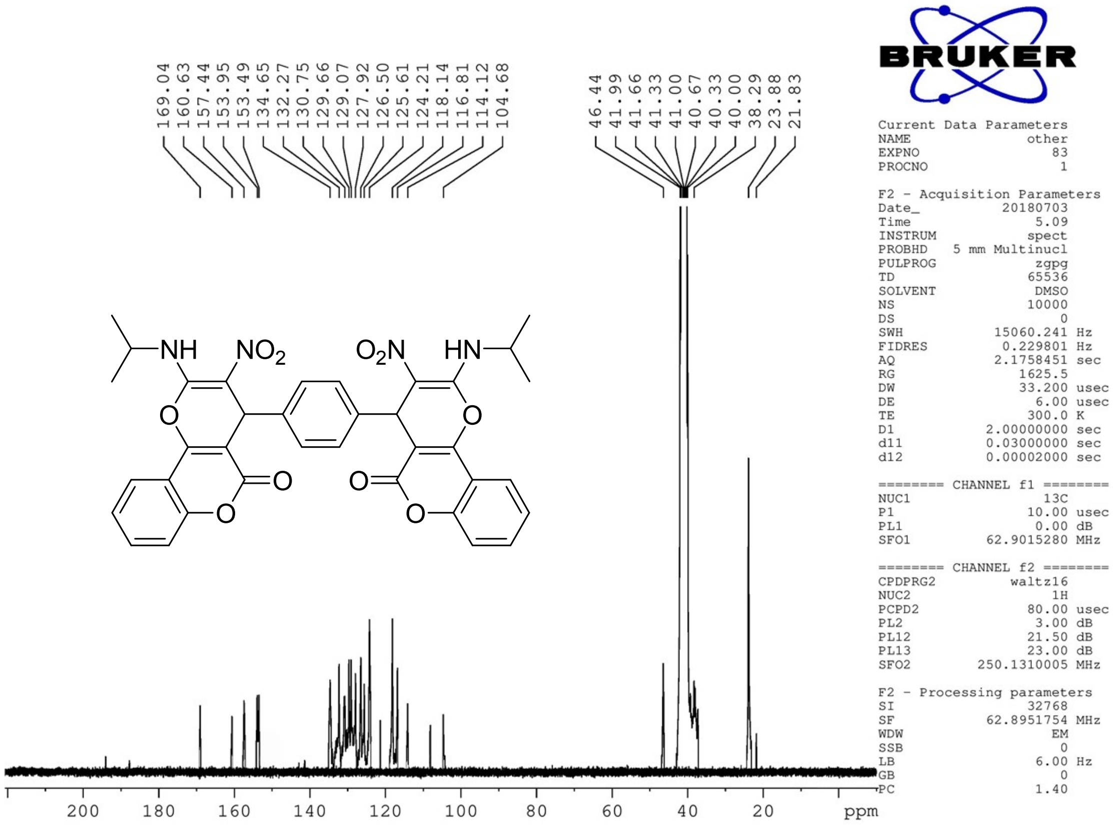
169.04
160.63
157.44
153.95
153.49
134.65
132.27
130.75
129.66
129.07
127.92
126.50
125.61
124.21
118.14
116.81
114.12
104.68
46.44
41.99
41.66
41.33
41.00
40.67
40.33
40.00
38.29
23.88
21.83
NH
NO2
O2N
HN
O
O
O
O
O
O
Current Data Parameters
NAME other
EXPNO 83
PROCNO 1
F2 - Acquisition Parameters
Date_ 20180703
Time 5.09
INSTRUM spect
PROBHD 5 mm Multinucl
PULPROG zgpg
TD 65536
SOLVENT DMSO
NS 10000
DS 0
SWH 15060.241 Hz
FIDRES 0.229801 Hz
AQ 2.1758451 sec
RG 1625.5
DW 33.200 usec
DE 6.00 usec
TE 300.0 K
D1 2.00000000 sec
d11 0.03000000 sec
d12 0.00002000 sec
======== CHANNEL f1 ========
NUC1 13C
P1 10.00 usec
PL1 0.00 dB
SFO1 62.9015280 MHz
======== CHANNEL f2 ========
CPDPRG2 waltz16
NUC2 1H
PCPD2 80.00 usec
PL2 3.00 dB
PL12 21.50 dB
PL13 23.00 dB
SFO2 250.1310005 MHz
F2 - Processing parameters
SI 32768
SF 62.8951754 MHz
WDW EM
SSB 0
LB 6.00 Hz
GB 0
PC 1.40
200
180
160
140
120
100
80
60
40
20
ppm

^{13}C NMR of 7e
